# CD151 drives cancer progression depending on integrin α3β1 through EGFR signaling in non-small cell lung cancer

**DOI:** 10.1186/s13046-021-01998-4

**Published:** 2021-06-09

**Authors:** Jianjie Zhu, Tingting Cai, Jieqi Zhou, Wenwen Du, Yuanyuan Zeng, Ting Liu, Yulong Fu, Yue Li, Qian Qian, Xiuwei H. Yang, Qinglin Li, Jian-an Huang, Zeyi Liu

**Affiliations:** 1 Department of Pulmonary and Critical Care Medicine, the First Affiliated Hospital of Soochow University, Suzhou, 215006 China; 2 Institute of Respiratory Diseases, Soochow University, 215006 Suzhou, China; 3 Suzhou Key Laboratory for Respiratory Diseases, 215006 Suzhou, China; 4 Department of Medicine, Division of Allergy and Clinical Immunology, National Jewish Health, Denver, 80206 USA; 5 Department of Pharmacology and Nutritional Sciences, Department of Molecular and Cellular Biochemistry, Markey Cancer Center, University of Kentucky, Lexington, KY 40506 USA; 6 Department of Traditional Chinese Medicine, Cancer Hospital of the University of Chinese Academy of Sciences, 310022 Hangzhou, People’s Republic of China

**Keywords:** CD151, Integrins, EGFR/ErbB2, Proliferation, NSCLC

## Abstract

**Background:**

Tetraspanins CD151, a transmembrane 4 superfamily protein, has been identified participating in the initiation of a variety of cancers. However, the precise function of CD151 in non-small cell lung cancer (NSCLC) remains unclear. Here, we addressed the pro-tumoral role of CD151 in NSCLC by targeting EGFR/ErbB2 which favors tumor proliferation, migration and invasion.

**Methods:**

First, the mRNA expression levels of CD151 in NSCLC tissues and cell lines were measured by RT-PCR. Meanwhile, CD151 and its associated proteins were analyzed by western blotting. The expression levels of CD151 in NSCLC samples and its paired adjacent lung tissues were then verified by Immunohistochemistry. The protein interactions are evaluated by co-immunoprecipitation. Flow cytometry was applied to cell cycle analysis. CCK-8, EdU Incorporation, and clonogenic assays were used to analyze cell viability. Wound healing, transwell migration, and matrigel invasion assays were utilized to assess the motility of tumor cells. To investigate the role of CD151 *in vivo*, lung carcinoma xenograft mouse model was applied.

**Results:**

High CD151 expression was identified in NSCLC tissues and cell lines, and its high expression was significantly associated with poor prognosis of NSCLC patients. Further, knockdown of CD151 *in vitro* inhibited tumor proliferation, migration, and invasion. Besides, inoculation of nude mice with CD151-overexpressing tumor cells exhibited substantial tumor proliferation compared to that in control mice which inoculated with vector-transfected tumor cells. Noteworthy, we found that overexpression of CD151 conferred cell migration and invasion by interacting with integrins. We next sought to demonstrate that CD151 regulated downstream signaling pathways via activation of EGFR/ErbB2 in NSCLC cells. Therefore, we infer that CD151 probably affects the sensitivity of NSCLC in response to anti-cancer drugs.

**Conclusions:**

Based on these results, we demonstrated a new mechanism of CD151-mediated tumor progression by targeting EGFR/ErbB2 signaling pathway, by which CD151 promotes NSCLC proliferation, migration, and invasion, which may considered as a potential target of NSCLC treatment.

**Supplementary Information:**

The online version contains supplementary material available at 10.1186/s13046-021-01998-4.

## Background

Lung cancer is the most frequent cause of cancer-related deaths worldwide [1, 2]. Non-small cell lung cancer (NSCLC) accounts for approximately 85 % of all lung cancers [3]. Although the advancement has been made in surgical procedure and chemical therapies, prognosis of NSCLC remains dismal [4]. Hence, exploring a better understanding of the mechanism underlying NSCLC progression and metastasis is extremely crucial.

Tetraspanins are four-transmembrane-spanning proteins containing short cytoplasmic N- and C-termini and one small and one large extracellular domain, which are highly expressed on cell surface or intracellular vesicle [5]. The most important character of tetraspanins is that they form complexes by interacting with each other and/or other transmembrane proteins, including integrins, RTK (EGFR and c-Met), cytoskeleton and cytosolic signal transduction molecule [6]. Therefore, tetraspanins are considered as regulators of cellular signaling and are often depicted as molecular facilitators [7].

Tetraspanins CD151, a transmembrane 4 superfamily protein, was identified as a positive effector associated with tumor development [8]. Recent studies have shown that CD151 expression was increased in breast, prostate, lung, colon, skin, and other cancers, and elevated CD151 expression was correlated with advanced stage and poor prognosis [9–11]. CD151 regulated laminin-binding integrins and control tumor cell migration and invasion through its effect on their adhesive and signaling functions [12]. While it has been recognized that high expression of CD151 was correlated with high grade and stage of NSCLC [13], the precise mechanism of CD151 in NSCLC is still unknown.

The purpose of this study was to investigate the role of CD151 in the progression and metastasis of NSCLC. To this end, we detected the expression of CD151 in tumor tissues and its paired adjacent tissues, and then analyzed its correlation with prognosis and pathological parameters. Further, the pro-tumoral effect of CD151 in NSCLC progression and metastasis was validated *in vivo* and in *vitro*. All together, our results indicated that CD151 plays an important role in tumor progression and metastasis of NSCLC.

## Materials and methods

### Cell culture

Human bronchial epithelial (16HBE) cell, human NSCLC cells A549, H1299, SPC-A1, H1650, Calu-3, 95 C, 95D (lung adenocarcinoma cell line) and H460 (giant-cell lung carcinoma cell line) were purchased from the Cell Bank of the Chinese Academy of Sciences (Shanghai, China). The cells were seeded and grown in RPMI 1640 medium (HyClone, South Logan, UT, USA), with 10 % fetal bovine serum (Gibco, Carlsbad, CA) and l-glutamine and antibiotics (Invitrogen, Carlsbad, CA, USA) at 37 °C in a humidified atmosphere containing 5 % CO_2_.

### NSCLC tissue samples

One hundred and seven paired NSCLC tissues and adjacent noncancerous lung tissues were collected after informed consent from patients in the First Affiliated Hospital of Soochow University between 2009 and 2015 (approval number/ID of the permission was 2009-157-1). Histological and pathological diagnostics for patients with NSCLC were evaluated according to the Revised International System for Staging Lung Cancer. The patients with NSCLC had received neither chemotherapy nor radiotherapy before tissue sampling. The demographic and clinical characteristics of NSCLC patients were summarized in Additional file 1: Table 1. Tissue samples were frozen rapidly and stored at − 80 °C in an ultra-deep freezer. This study was approved by the Academic Advisory Board of Soochow University (number/ID of the permission was 2009-157-1).

### Immunohistochemical assay

Tissue Microarray Matched pairs of NSCLC samples and adjacent lung tissues were used for the construction of tissue microarray (Outdo Biotech, Shanghai, China) as previously described [14]. In brief, the sections were incubated with CD151 antibody (diluted to 1:50; Santacruz, sc-271,216) overnight at 4 °C, and then incubated with the corresponding biotinylated secondary antibodies. The reactions were developed using the DAB Kit (BD Bioscience, San Jose, CA, USA), and the sections were counterstained with hematoxylin.

The CD151 expression levels were determined as bellow: the expression levels for each sample which represented by score (intensity × positive rate), as reported previously [15]. The scoring of immunostaining was evaluated on the basis of staining intensity and percentages of three positively stained areas at random by two pathologists in a double blinded manner. Briefly, the proportion of positive cells in each specimen was quantitatively evaluated and scored as follows: 0, Staining in 0 % of the cells examined; 1, staining in 0.01-10 % of the cells examined; 2, staining in 10.01-50 % of the cells examined; 3, staining in 50.01-75 % of the cells examined; and 4, staining in > 75 % of the cells examined. The staining intensity was graded as follows: 0, No signal; 1, weak; 2, moderate; and 3, strong. The histological score for each section was computed using the following formula: Histological score = proportion score x intensity score. A total score with a possible range of 0–12 was calculated and graded as follows: Negative (-; score, 0), weak (+; score, 1–4), moderate (++; score, 5–8) or strong (+++; score, 9–12). Scores of - and + were considered to indicate low expression levels, whereas scores of + + and +++ were considered to indicate high expression levels.

### RNA extraction and quantitative real-time PCR analysis

Total RNA was extracted from cells and tissues using RNAiso Plus (TaKaRa, Osaka, Japan) according to the manufacturer’s protocol. Synthesis of cDNA with reverse transcriptase (RT) was performed with Reverse Transcriptase M-MLV (TaKaRa, Osaka, Japan). Primer sequences for mRNA detection are as follows: CD151: 5′-GGGCCACTTGCATGTTCGT-3′ (forward), 5′-CAGGTTCCACTTGAGCTTGTTCAC-3′(reverse); β-actin: 5′-CACAGAGCCTCGCCTTTGCC-3′ (forward), 5′-ACCCATGCCCACCATCACG-3′ (reverse). Real-time PCR analysis was performed using SYBR Premix Ex TaqTM (TaKaRa, Osaka, Japan) and ABI Step One Plus Real-Time PCR system (Applied Biosystems, Foster City, CA, USA). Ct values of CD151 mRNA were equilibrated to β-actin, which were used as internal controls. The ^ΔΔ^Ctmethod was applied to calculated the relative expression.

### Generation of stable cell lines overexpressing CD151 and establishment of CD151-silenced stable cell lines

To generate A549 and H1299 cells in which CD151 can stably overexpress, we subcloned a ~ 800 bp coding sequence (GenBank Accession number NM_004357.5) of CD151 into a pGMLV-CMV-MCS-EF1 vector using endonucleases EcoRI and XhoI for expression via Lenti-X lentiviral expression system (Clontech, Mountain View, CA, USA). Then the CD151 expression construct was co-transfected with packaging plasmids into human embryonic kidney 293 T cells using Lipofectamine 2000 (Invitrogen). The empty vector was served as a negative control. Human embryonic kidney 293 T cells were cultured in Dulbecco’s modified Eagle’s medium with 10 % fetal bovine serum at 37 °C in a humidified 5 % CO_2_ incubator for 48 h. After the incubation, the packaged lentiviruses were collected and used to infect A549 and H1299 cells. After 2 days, stable cells were selected with 2 µg/ml of puromycin (Sigma-Aldrich, St Louis, MO, USA). The coding sequence region of CD151 was amplified using the following primers: forward, 5′-CCGGAATTCCCAGGATGGGTGAGTTCAAC-3′(EcoRI); reverse, 5′-CCGCTCGAGGGCAG GGTCAGTAGTGCTCC-3′ (XhoI). To establish stable A549 and H1299 cell lines in which CD151 is silenced, two DNA fragments (CD151 shRNA-1, 5′-GCCCTCAAGAGTGACTACATT TCAAGAGAATGTAGTCACTCTTGAGGGTTTTTT-3′; and CD151 shRNA-2, 5′-AGCTCAAG GAGAACCTGAATTCAAGAGATCAGGTTCCTTGAGCTTTTTTT-3′) were subcloned a lentiviral vector pGMLV-SC5 (Genomeditech, Shanghai, China) with endonucleases BamHI and EcoRI. A scrambled sequence (underscored) of CD151 shRNA, which was served as negative control, was as follows: 5′-ATCGACTAGCCACTTAGACTTCAAGAGGTCTAAGTGGCTAGT CGATTTTTTTT-3′. Then, the CD151-silenced construct or negative control was co-transfected with packaging plasmids into human embryonic kidney 293 T cells using Lipofectamine 2000 (Invitrogen). Forty-eight hours later, A549 and H1299 cells were infected with the packaged lentiviruses and cultured for 2 days, and stable cell lines were selected with 2 µg/ml of puromycin.

### Antibody and proteome arrays

For the RTK activation study, antibody arrays by Raybio (Catalog: AAH-PRTK-G1) were used according to the manufacturer’s protocol, it is specifically designed for simultaneously identifying the relative levels of phosphorylation of 71 different Human Receptor Tyrosine Kinases (RTKs) in cell lysate. In brief, cell lysates from A549 stable cells and control cells were collected and incubated with the blocked Glass Chip for overnight at 4 ℃ with gentle shaking. After development, the laser signals were captured and analyzed using the GenePix 4000B Microarray Scanner (Molecular Devices, CA, United States). For the soluble receptors and related proteins analysis, we used the Proteome Profiler^TM^Array (R&D Systems, catalog number ARY012) and processed according to the manufacturer’s instructions. Protein lysates were incubated with the array membrane and pixel densities on developed X-ray film can be collected and analyzed using a transmission mode scanner and image analysis software.

### Western blot assay

The tissues and transfected cells were lysed using 1×RIPA buffer with protease inhibitors and phosphatase inhibitors(Apexbio), shaken on ice for 30 min, and centrifuged at 4 °C, 12,000 g for 15 min. The extracted proteins were separated using a 10 % sodium dodecyl sulphate-polyacrylaminde gel electrophoresis(SDS-PAGE) and subsequently transferred to NC membranes (Millipore, Billerica, MA, USA). Antibodies employed in the analysis were as follows: anti-CD151 (sc-271216) and anti-EGFR (sc-373746) (Santa Cruz, CA,USA);anti-Integrin α3 (ab242196), anti-Integrin α6 (ab20142), anti-Integrin β1 (ab52971) (Abcam, Cambridge, UK);p-ErbB2 (Sigma-Aldrich; Merck KGaA, SAB4300061); anti-p-EGFR (3777), anti-ErbB2 (4290), anti-p-FAK (8556), anti-FAK (13,009), anti-p-AKT (4060), anti-AKT(4691), anti-p-ERK (4370), anti-ERK (4695), anti-p-Src (2101), anti-Src (2109), anti-CyclinD1 (2978), anti-MMP2 (13132), anti-MMP9 (13,667) were all purchased form Cell Signaling Technology (Danvers, MA, USA).Anti-β-actin (CW0096M) and anti-mouse (CW0102) or anti-rabbit (CW0103) secondary antibodies were purchased from Cowin.

### Co-immunoprecipitation (co-ip) assay

NSCLC cells were cultured in a 100mm plate to 95– 100 % confluence. Then, the cells in each dish were washed twice with cold phosphate-buffered saline (PBS), collected by scraping, and lysed with 1ml of modified RIPA buffer (Cell Signaling Technology, Danvers, MA, USA) containing protease and phosphatase inhibitor cocktail (Sigma-Aldrich, St. Louis, MO, USA) for 30 min. Cell lysates were collected by centrifugation at 10, 000×g at 4 °C for 30 min. Clear lysates were pre-cleared by the addition of 50 µl of protein G bead slurry and incubated at 4 °C overnight with rotation. Supernatants were transferred to a new Eppendorf tube and incubated with 1 µg of mouse anti-CD151 (Santacruz, sc218216) antibody with rotation overnight in a cold room; this step was followed by an additional incubation for 3–4 h with protein G beads. The beads were washed three times with RIPA buffer and then boiled in 2× SDS protein loading buffer for 5 min. Samples (20 µl) were loaded on SDS-PAGE gels for western blot analysis.

### Immunofluorescence staining

Cultured cells were fixed with 4 % paraformaldehyde for 15 min at room temperature, permeablized with triton (0.1 % in TBS) for 30 min and blocked with 5 % BSA in PBS for 1 h at room temperature. Cells were then incubated overnight at 4℃ with anti-CD151 (Santa Cruz, sc218216), anti-ITGα3 (Abcam, Ab242196), anti-p-EGFR (Abcam, ab40815). The corresponding secondary antibodies tagged with Cy3 and FITC were used (1:500, Beyotime Biotechnology). Finally, the samples were incubated in DAPI for 10 min (Life Technologies) for nuclear counterstain. Images were acquired using a Leica SP8 confocal microscope with optimal setting for the fluorescent markers used.

### Transfection

A549 and H1299 stable cells were seeded in 6-well plates. When cells had reached 40–60 % confluence, we performed transfection in accordance with the manufacturer’s instructions using jetPRIME reagent (Invitrogen). Cells were collected at 48–72 h after transfection for further experiments. The ITGA3, ITGA6 and ITGB1 siRNA and corresponding control were purchased from GenePharmacompany (Suzhou, China). The target siRNA sequences were as follows: ITGA3 siRNA: 5′-UUACAGAGACUUUGACCGATT-3′; ITGA6 siRNA: 5′-CAAACAGCUCAUAU UGAUTT-3′; ITGB1 siRNA: 5′-CAGCCCAUUUAGCUAAAAT-3′.

### Cell proliferation analysis

Cell proliferation was determined by using the Cell Counting Kit-8 assay kit (Dojindo, Shanghai, China). The A549 and H1299 stable cells were seeded in 96-well plates at 2 × 10^3^ cells per well and further grown in normal culture condition for 24, 48 and 72 h. Cell viability was measured according to manufacturer’s instructions. The experiment was performed in triplicate.

### EdU incorporation assay

Cell proliferation also was determined using EdU (5-ethynyl-2-deoxyuridine) assay (Ribobio). Briefly, 3 × 10^4^/mL of A549 and H1299 stable cells were plated in 96-well plates, then after 48 h were exposed to EdU for 2 h. Subsequently, cells were fixed with 4 % formaldehyde 30 min at room temperature. After neutralization with glycine and washing, cells were treated with 0.5 % TritonX-100 for 30 min and reacted with Apollo® reaction cocktail for 30 min. Nuclei were stained with Hoechst 33,342. The EdU-positive cells were visualized under a fluorescent microscope (Olympus) and counted with Image J software.

### Cell cycle analysis

According to the instructions of the Cell Cycle Analysis Kit (Beyotime, Shanghai, China), cells were cultured in 6-well plates for 72 h. The cells were then collected, washed with cold phosphate-buffered saline (PBS), fixed in 70 % ethanol at 4 °C for 24 h, washed with cold PBS again and stained in a propidium iodide (PI) / RNaseA mixture. Next, the cells were kept in the dark at 37 °C for 30 min and 10,000 + cells were analyzed per sample using a fluorescence-activated cell sorting (FACS) Caliber system (Beckman Coulter, Brea, CA, USA).

### Migration and invasion assays

Transwell inserts in size of 8.0 μm pore (Corning, NewYork, NY, USA) were used for performing cell migration and invasion assays. For migration assay, 800 µl RPMI-1640 medium with 10 %FBS was added into each lower chamber of a transwell insert. Briefly, the stable cells were trypsinized, and then 5 × 10^4^ cells with medium containing 1 % FBS were seeded into the upper chamber and incubated at 37℃ for 24 h in a humidified incubator. Furtherly, the cells migrated onto the lower surface of the insert were fixed with 100 % methanol for 20 min, air-dried for 10 min, stained with 0.1 % crystal violet overnight and washed with 1×PBS for two times. Lastly, the cells were photographed and counted. For invasion assay, the inserts were coated with Matrigel matrix (BD Science, Sparks, MD, USA) diluted in serum-free medium, then incubated at 37℃ for 2 h, remaining procedures were conducted similar to migration assay. Each experiment was performed in triplicate.

### Wound healing assay

Seed A549 and H1299 stable cells into 6-well tissue culture plate at a density, then they should reach ~ 70–80 % confluence as a monolayer. The monolayer was gently and slowly scratched using a fresh 10-µl pipette tip across the centre of the well, aiming for a resulting gap distance equal to the outer diameter of the end of the tip. Another scratch was made perpendicular to the first to create a cross in each well. Detached cells were then removed by two gentle washes with 1 × PBS. The well was replenished with fresh medium, and cells were cultured for an additional 24 h. Cells were observed and imaged under a microscope (CKX41, Olympus) at the same magnification and settings. The width of the gap was evaluated quantitatively using Photoshop.

### Animal experiments

Female BALB/c athymic nude mice (4–6 weeks old and weighing 16–20 g) were purchased from the Experimental Animal Center of Soochow University and bred under pathogen-free conditions. All the animal experiments were carried out in accordance with the Guide for the Care and Use of Experimental Animals Center of Soochow University. To establish the lung carcinoma xenograft model, 1 × 10^6^ A549/sh-NC, A549/sh-CD151, A549/vector or A549/CD151 cells were suspended in 0.1 ml of RPMI 1640 medium without fetal bovine serum and inoculated subcutaneously into the flanks of nude mice, which were randomly divided into two groups (4 mice in each group). Tumor volume (V) was determined by measuring the length (L) and width (W) with a vernier caliper and applying the following formula: V = (L ×W^2^) × 0.5.

### Statistical analysis

All statistical analyses were performed using Graphpad Prism 7.0 (Graphpad, San Diego, CA, USA) and the SPSS 17.0 software. Data were presented as the mean ± standard error of the mean (SEM). The quantitative data were analyzed using an independent-samples t-test for comparison between two groups. Comparisons among three or more than three groups were performed using one-way ANOVA test followed by Bonferroni’s post hoc test. The expression level of CD151 between NSCLC tissues (T) and adjacent noncancerous lung tissues (N) were analyzed using the paired-samples t-test. The survival differences among groups were calculated using the Kaplan-Meier method with a log-rank test. Statistical significance was identified with *P* < 0.05.

## Results

### CD151 is highly expressed in NSCLC tissues and cell lines and its elevated expression is correlated with poor prognosis

**Fig. 1:**
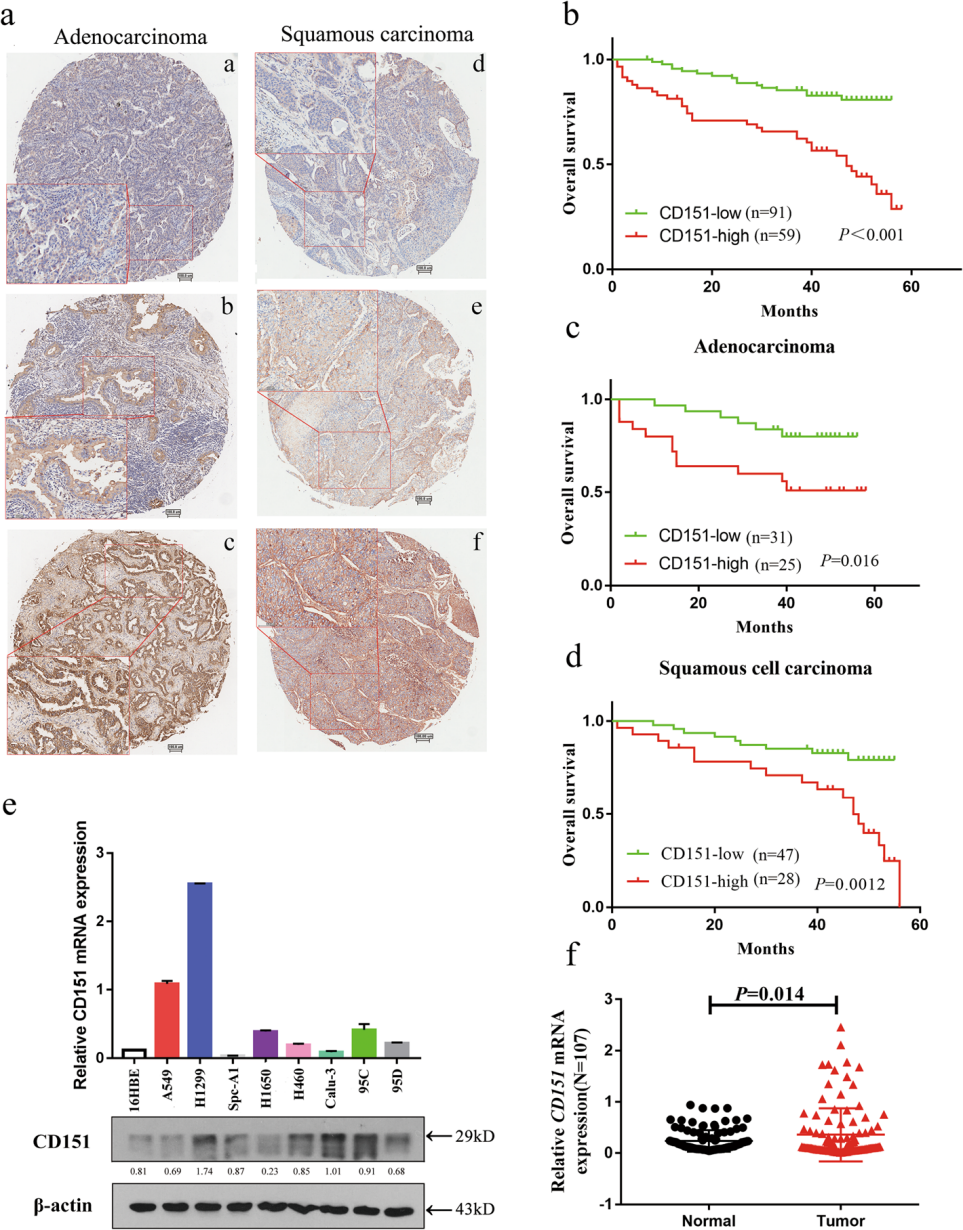
CD151 expression is up-regulated in NSCLC tissues and cell lines.** a** NSCLC samples were immunostained with anti-CD151antibody. Representative adenocarcinomas sample **(a–c)** and squamous cell carcinomas sample **(d–f)** are shown. **b** Effect of the CD151 expression level on overall survival in 150 lung cancer patients was analyzed. **c-d** Kaplan-Meier analysis of overall survival for CD151 expression in adenocarcinomas or squamous cell carcinomas samples. **e** CD151 mRNA and protein expression in human NSCLC cell lines were analyzed by RT-PCR and Western blot, respectively. **f** CD151 mRNA levels in 107 NSCLC tissues and paired noncancerous lung tissues. Bars represent mean ± SEM from three independent experiments. *P < 0.05; **P < 0.01; ***P < 0.001

Immunohistochemistry (IHC) analysis was carried out to evaluate CD151 protein level in 150 paired NSCLC tissues. Data showed that increased CD151 expression was detected in 39.3 % NSCLCs (59/150), including 36 % SCCs (27/75) and 46.4 % ADCs (26/56) (Fig. 1a). The summary of patient characteristics and correlation coefficients with CD151 expression are listed in Additional file 1: Table S1. Our data showed that enlarged CD151 expression was significantly associated with T status (*P* = 0.014), N status (*P* < 0.001), Clinical stage (*P* < 0.001) and pathological grade (*P* = 0.002). Of note, there was no significant correlation between CD151 expression and gender, age or histological characteristics. We further analyzed the prognostic relevance of CD151 expression and overall survival among NSCLC patients. Our data showed that patients with high CD151 expression level was associated with poorer overall survival among 150 NSCLC patients (Fig. 1b), which was consistent with the results in NSCLC subsets, including adenocarcinoma and squamous carcinoma (Fig. 1c, d). RT-PCR analysis also showed up-regulation of CD151 mRNA expression in 107 paired NSCLC tissues compared to adjacent tissues (Fig. 1f). Of note, there was no significant differences found in CD151 mRNA level in terms of patients’ age, gender, smoking habits, degree of differentiation, TNM stage and lymph node infiltration; however, significant differences were observed in terms of histological characteristics and distant metastasis (Additional file 2: Table S2). Consistently, we also found that CD151 was up-regulated in NSCLC cell lines in both mRNA and protein level (Fig. 1e). Collectively, our data indicated the clinical significance of CD151 which may play an important role in NSCLC carcinogenesis.

### Knockdown of CD151 inhibits NSCLC cells proliferation, migration and invasion *in vitro*

**Fig. 2:**
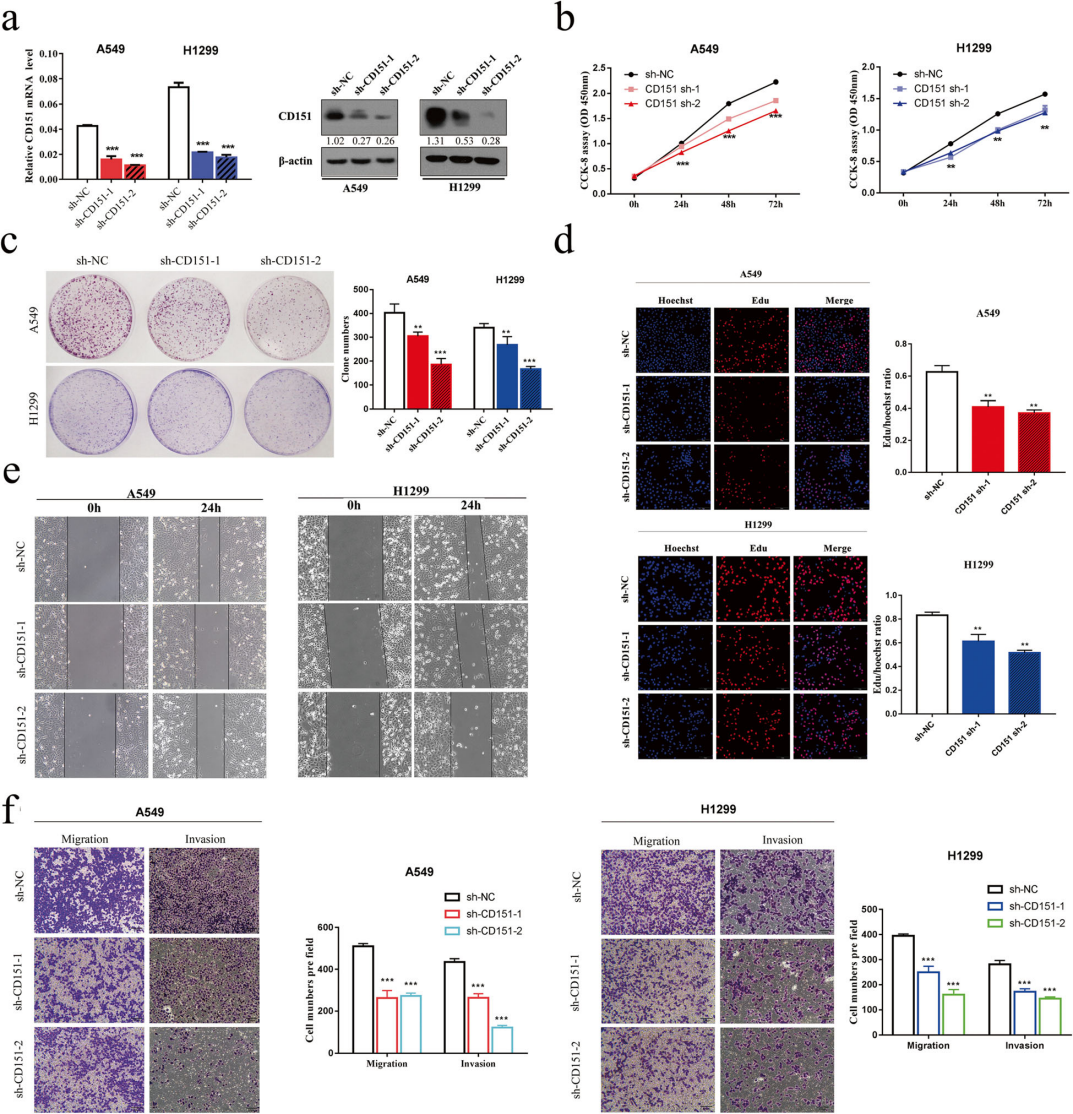
Silencing of CD151 inhibits NSCLC cell proliferation, migration and invasion. **a** CD151 mRNA and protein levels in NSCLC cell lines which transfected with either CD151 shRNAs (sh-CD151-1 and sh-CD151-2) or negative control (sh-NC). **b** CCK-8 assay of cell viability in NSCLC cell lines. **c** Representative images of clonogenic analysis of cell proliferation in NSCLC cells. Bar charts showed clonogenic growth of NSCLC cells. **d** EdU incorporation assay for cell proliferation (*n* = 3). EdU (red), DAPI (blue). Scale bar = 200 μm. **e** Wound healing assay was performed to observe the role of CD151 in A549 and H1299 cells. **f** Representative images of the transwell assay results for cell migration and invasion in A549 and H1299 cells (sh-CD151 compared with sh-NC). β-actin was used as the internal control. Bars represent mean ± SD from three independent experiments. Significant differences compared with the control: * *P* < 0.05; **P < 0.01; ***P < 0.001

To determine the role of CD151 in NSCLC, two NSCLC cell lines A549 and H1299 were chosen to construct stable CD151 knockdown cell lines. CD151 expression was significantly down-regulated in both mRNA and protein level after the stable transfection with two distinct short hairpin RNAs (shRNAs) individually (Fig. 2a). Next, CCK-8 and clonogenic assays were applied to show that CD151 knockdown can inhibit cell proliferation (Fig. 2b, c). We also confirmed the findings via EdU assay (Fig. 2d). Additionally, cell numbers in S phase were reduced accompanied by increased cell numbers in G0/G1 phase in CD151 knockdown cell lines, implying that CD151 may affect cell proliferation via regulating cell cycle (Additional file3: Fig. S1). We further assessed the role of CD151 in regulating cell migration and invasion. Wound healing assay confirmed that the migratory ability was suppressed in both A549 and H1299 cells after silencing CD151 expression (Fig. 2e). Transwell assay showed less cells were migrated through the inserts in the presence of intrinsic CD151 knockdown (Fig. 2f).

### CD151 overexpression promotes NSCLC cells proliferation, migration and invasion

**Fig. 3:**
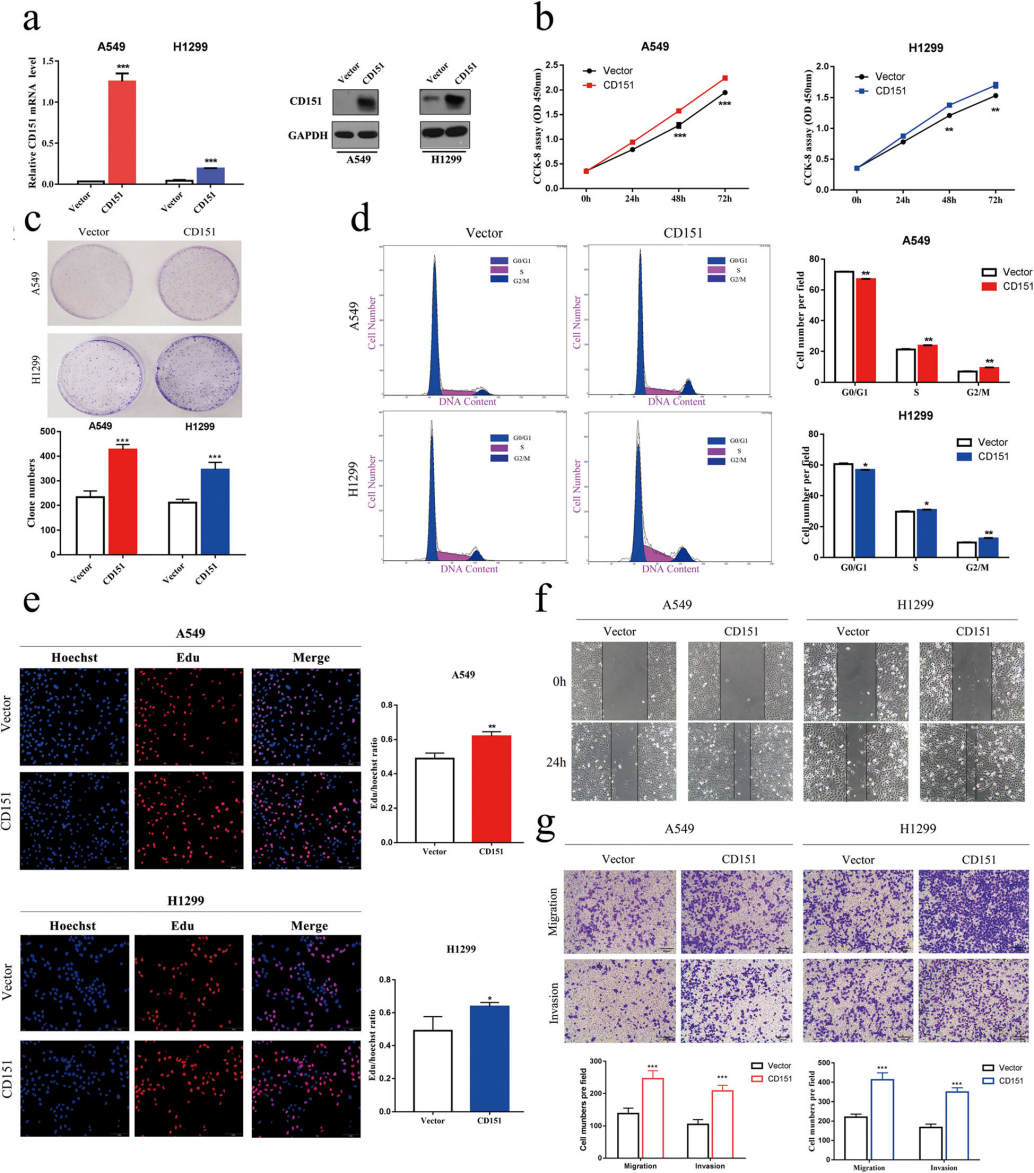
Overexpression of CD151 promotes NSCLC cell proliferation, migration and invasion.** a** CD151 mRNA and protein levels in CD151-overexpressed NSCLC cell lines. **b** CCK-8 assay of cell viability in NSCLC cell lines. **c** Representative images of clonogenic analysis of cell proliferation in NSCLC cells. Bar charts showed clonogenic growth of NSCLC cells. **d** Flow cytometry analysis of cell cycle of NSCLC cell lines (CD151-overexpressed cells vs. Vector cells). Cells were harvested at 72 h after stained with propidium iodide. **e** EdU incorporation assay for cell proliferation(*n* = 3). EdU (red), DAPI (blue). Scale bar = 200 μm. **f** Wound healing assay was performed to observe the role of CD151 in A549 and H1299 cells. **g** Representative images of the transwell assay results for cell migration and invasion in A549 and H1299 cells (CD151-overexpressed compared with vector). β-actin was used as the internal control. Bars represent mean ± SD from three independent experiments. Significant differences compared with the control: * *P* < 0.05; **P < 0.01; ***P < 0.001

Besides loss-of-function experiments, we also assessed CD151 overexpression in regulating NSCLC cell proliferation, migration and invasion. CD151 expression was confirmed to be significantly up-regulated via RT-PCR and western blot assay (Fig. 3a). We demonstrated that CD151 overexpression promotes cell proliferation via CCK-8 and clonogenic assays (Fig. 3b, c). EdU assay showed that the percentage of EdU positive cells is higher in CD151 overexpressed group compared to control group (Fig. 3e). The cell cycle assay confirmed that increased cell number was observed in S phase along with decreased cell number in G0/G1 phase in CD151 overexpressed cell lines (Fig. 3d). In addition, data from wound healing assay and transwell assay showed that CD151 overexpression enhanced the ability of cell migration and invasion (Fig. 3f, g). All above findings showed that CD151 promotes NSCLC cell proliferation, partially through regulating cell cycle, as well as migration and invasion.

### CD151 and Integrin α3β1 were highly correlated

**Fig. 4:**
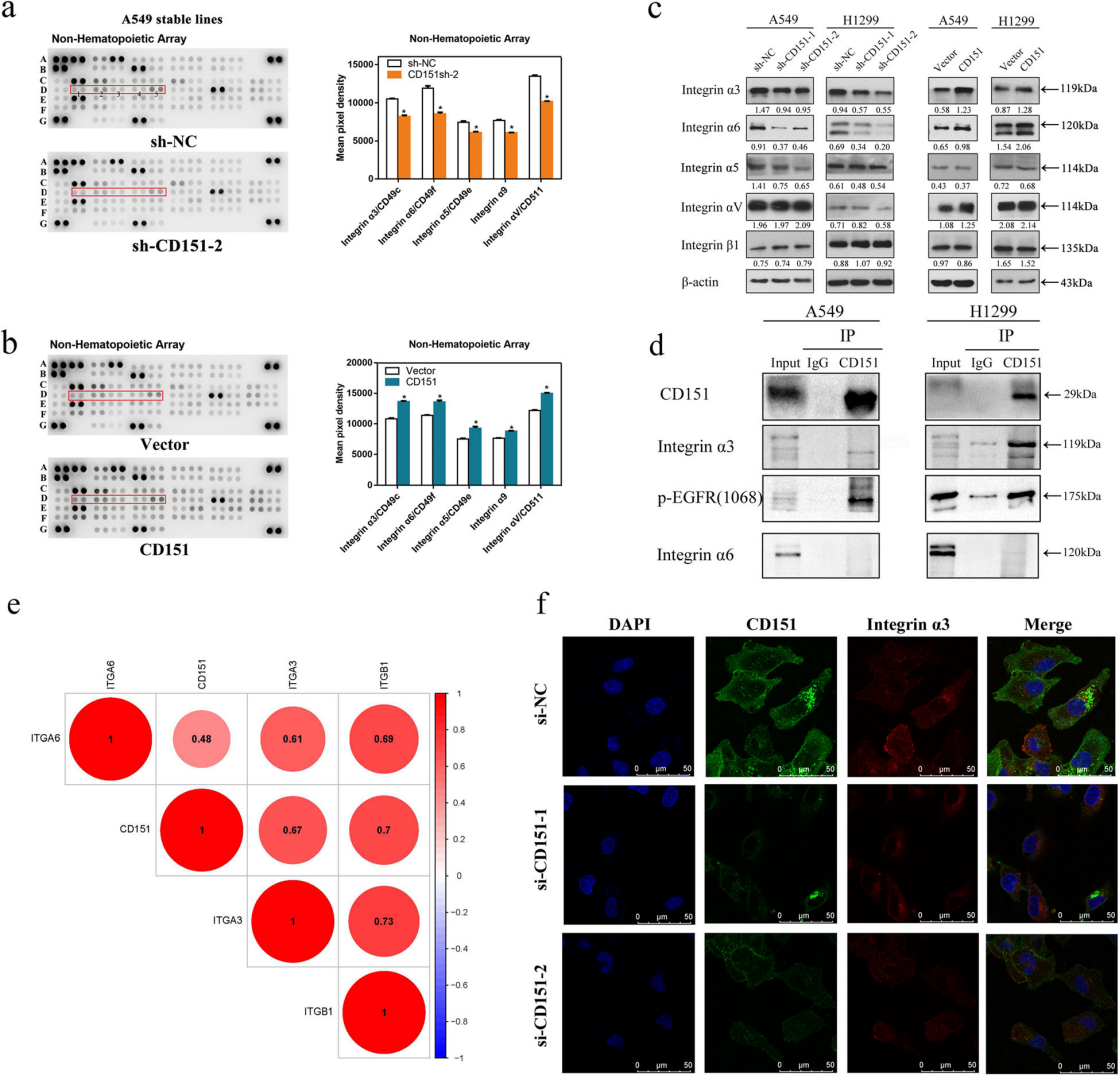
CD151 and integrin α3β1 were highcorrelated. **a-b** Human Soluble Receptor Antibody Array analysis of stable A549 cells in which CD151 either silenced or overexpressed. The list of up-regulated proteins in CD151-overexpressed A549 cells and the down-regulated proteins in CD151-silenced A549 cells are also shown. **c** The integrins were verified in A549 and H1299 stable cells using western blot. **d** Co-immunoprecipitation of CD151 and integrin and p-EGFR are shown. Protein were immunoprecipitated and detected from lysates of A549 and H1299 cells using a specific monoclonal antibody. **e** Data from CPTA database showed the correlation of CD151 and integrin. **f** Immunofluorescence staining of CD151 and integrin α3 co-expression in CD151-knockdown cells compared to control cells (Scale bar: 5 μm). *P < 0.05; **P < 0.01; ***P < 0.001

CD151 has been suggested to regulate cell adhesion through its association with laminin-binding integrins; however, its precise function in NSCLC remains unclarified. To further investigate mechanisms, we analyzed cell adhension-associated proteomes and secretomes in both CD151 knockdown and overexpression cell lines using Proteome Profiler Array. The immunoblot membranes and quantified expression levels of proteomes and secretomes are showed in Fig. 4a and b, antibody map and related data were provided in Additional file 4: Fig. S2, Additional file 5: Fig. S3a, 3b, Additional file 6: Table S3(Part N and Part C). Data obtained from TCGA database (https://portal.gdc.cancer.gov/) were analyzed to explore the correlation between CD151 and integrin α3/α6/β1 mRNA levels in 103 normal tissues and 999 NSCLC tissues (Additional file 7: Fig. S4a-c) and 188 lung cancer cell lines (Additional file 7: Fig. S4d-f). Extracted data showed that integrin α family members were significantly changed through either CD151 knockdown or overexpression. On the contrary, the integrin β members were not changed. Consistently, western blot analysis confirmed these findings (Fig. 4c). We further investigated complexes of CD151 and integrins or p-EGFR using co-immunoprecipitation, the results showed that CD151 directly bound to integrin α3 in NSCLC (Fig. 4d), however, the combination of CD151 and integrin α6/β1 were likely to be indirected (Fig. 4d and Additional file 7: Fig. S4h). Indeed, there was a study reported integrin α3/β1–CD151 complex can regulate dimerization of ErbB2, but not integrin α6/β1–CD151 complex [16]. Data from CPTA database (https://cptac-data-portal.georgetown.edu/cptacPublic/) also confirmed the correlation of CD151 and integrin (Fig. 4e). Based on the above, we proved that interfering CD151 reduced the expression of integrin α3 (Fig. 4f). All above findings showed that CD151 and integrin α3/β1 are related.

### CD151 promotes NSCLC proliferation and metastasis via activating EGFR and ErbB2 signaling pathways

**Fig. 5:**
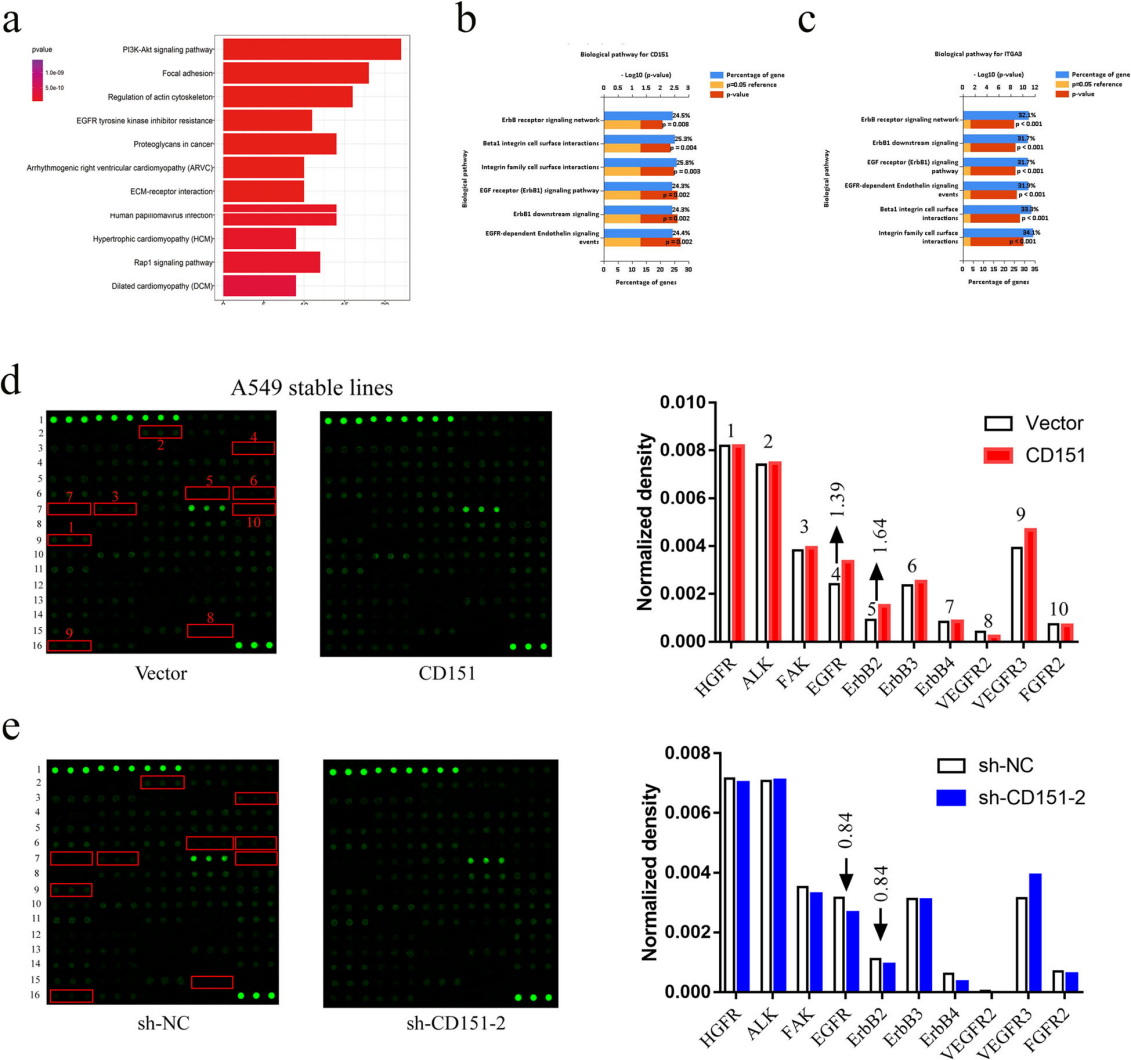
CD151 accosiated with ErbB signaling pathway. **a** Pathway analysis was used to identify the significant pathway of the differential genes according to the KEGG database. We used the Fisher’s exact test to select the significant pathways, and the threshold of significance was defined by p-value and FDR. **b-c** Data from TCGA database. **d-e** Total protein lysates from A549 stable cells were analyzed using an antibody array against 71 unique RTKs (array map provided in Figure S5). *P < 0.05; **P < 0.01; ***P < 0.001

**Fig. 6:**
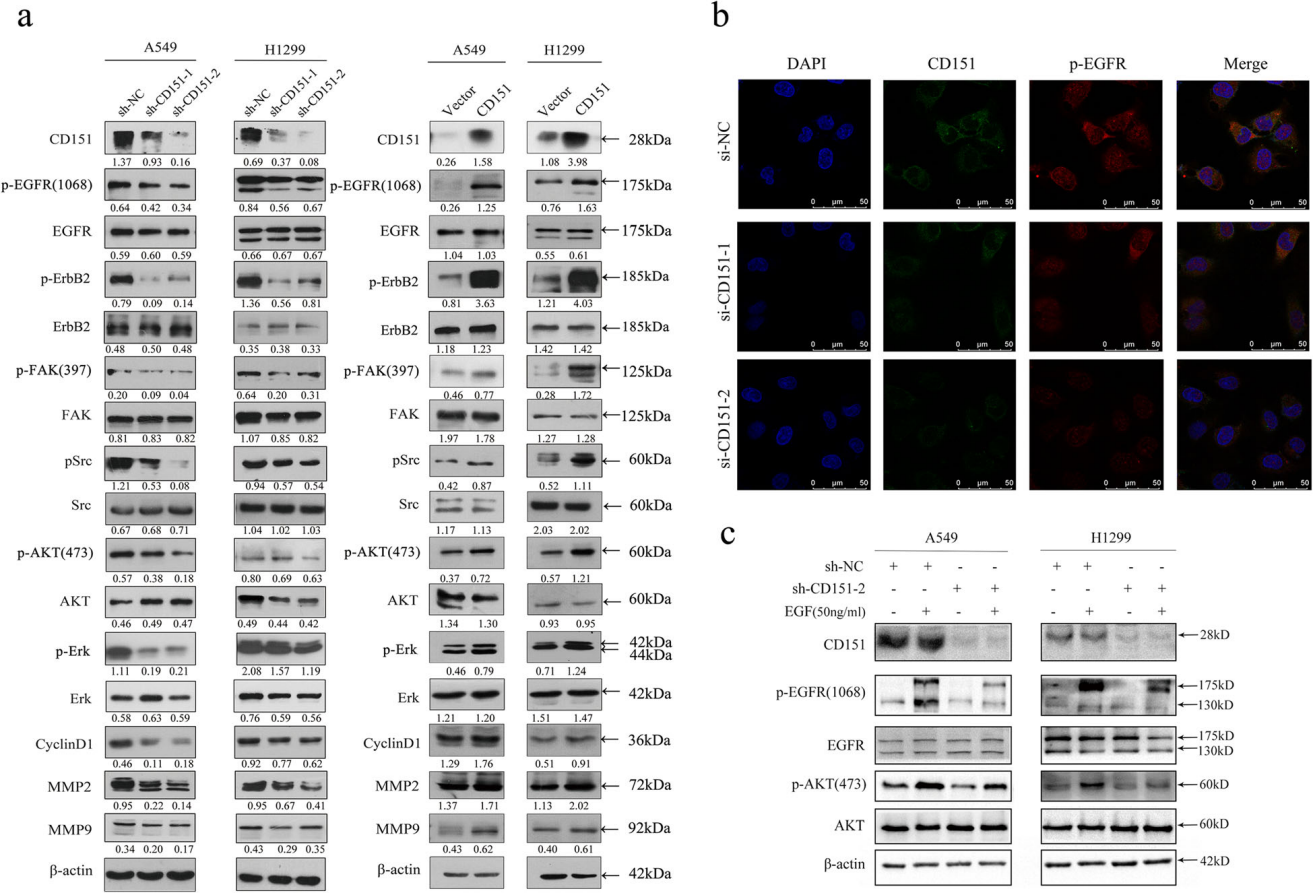
Abnormal expression of CD151 disrupts EGF signalling. **a** Western blot assay of CD151, p-EGFR, p-ErbB2, p-FAK, p-Src, p-AKT, p-Erk, Cyclin D1, MMP2 and MMP9 expression in stable CD151 knockdown or overexpressed cells when compared to control cells. β-actin was used as a loading control. **b** Immunofluorescence staining of CD151 and integrin p-EGFR co-expression in CD151-knockdown cells compared to control cells (Scale bar: 5 μm). **c** Control and CD151-knockdown cells were treated with EGF (50ng/ml) for 1 h, and the expression levels of various proteins were then measured by western blot analysis. β-actin was used as the internal control. Bars represent mean ± SD from three independent experiments. *P < 0.05; **P < 0.01; ***P < 0.001

To clarify the mechanism underlying CD151-mediated cell proliferation and metastasis, GO analysis, first of all, indicated that the PI3K-Akt and EGFR signaling pathways are important in CD151-mediated NSCLC cell proliferation, metastasis which is consistent with our results (Fig. 5a). Then, we exported non-small cell lung cancer mRNA expression data from TCGA database, and performed co-expression analysis with CD151 and ITGA3 co-expression coefficient of 0.3, *P* < 0.05, and input the selected genes into FunRich software (version 3.1.3) for functional analysis, it can be seen that most of the genes are clustered in EGFR related pathways (Fig. 5b, c). The Human RTK Phosphorylation Antibody Array G-series1 was also performed (Fig. 5d and e, antibody map provided in Additional file 8: Fig. S5 and Additional file 9: Table S4). The results showed that phosphorylation of EGFR and ErbB2 exhibited the largest fold changes among selected proteins due to CD151 expression manipulation (Fig. 5d, e). We therefore examined the EGFR/ErbB2 pathways as a candidate critical signaling pathway in our study. Western blot assay confirmed that the phosphorylation levels of EGFR and ErbB2 were down-regulated when knocking down CD151 and up-regulated when overexpressing CD151 in NSCLC cells. Sequentially, we showed that the downstream p-FAK, p-Src, p-AKT, p-Erk and the cell cycle associated cyclin D1 were down-regulated, while the total FAK, SRC, AKT and ERK unchanged. Moreover, the expression of MMP2 and MMP9, tumor metastasis-associated molecules, were reduced after silencing CD151 expression (Fig. 6a). By contrast, ectopic expression of CD151 enhanced p-EGFR, p-ErbB2, p-FAK, p-Src, p-AKT, p-Erk, Cyclin D1, MMP2 and MMP9 expression level as indicated above (Fig. 6a). Furthermore, immunofluorescence assays showed that CD151 and p-EGFR were co-expressed, when CD151 were interfered, the expression of p-EGFR were also decreased (Fig. 6b). In order to further illustrate the combination between CD151 and p-EGFR, we used the exogenous EGF in both control and CD151 knockdown cell lines, we found that the protein level of p-EGFR and p-AKT induced by EGF were significantly inhibited by CD151 knockdown (Fig. 6c). Collectively, these results demonstrated a crucial role of EGFR/ErbB2 signaling in CD151-mediated NSCLC cell proliferation and metastasis *in vitro*.

### Integrin signaling is involved in CD151-induced EGFR and ErbB2 pathways activation

**Fig. 7:**
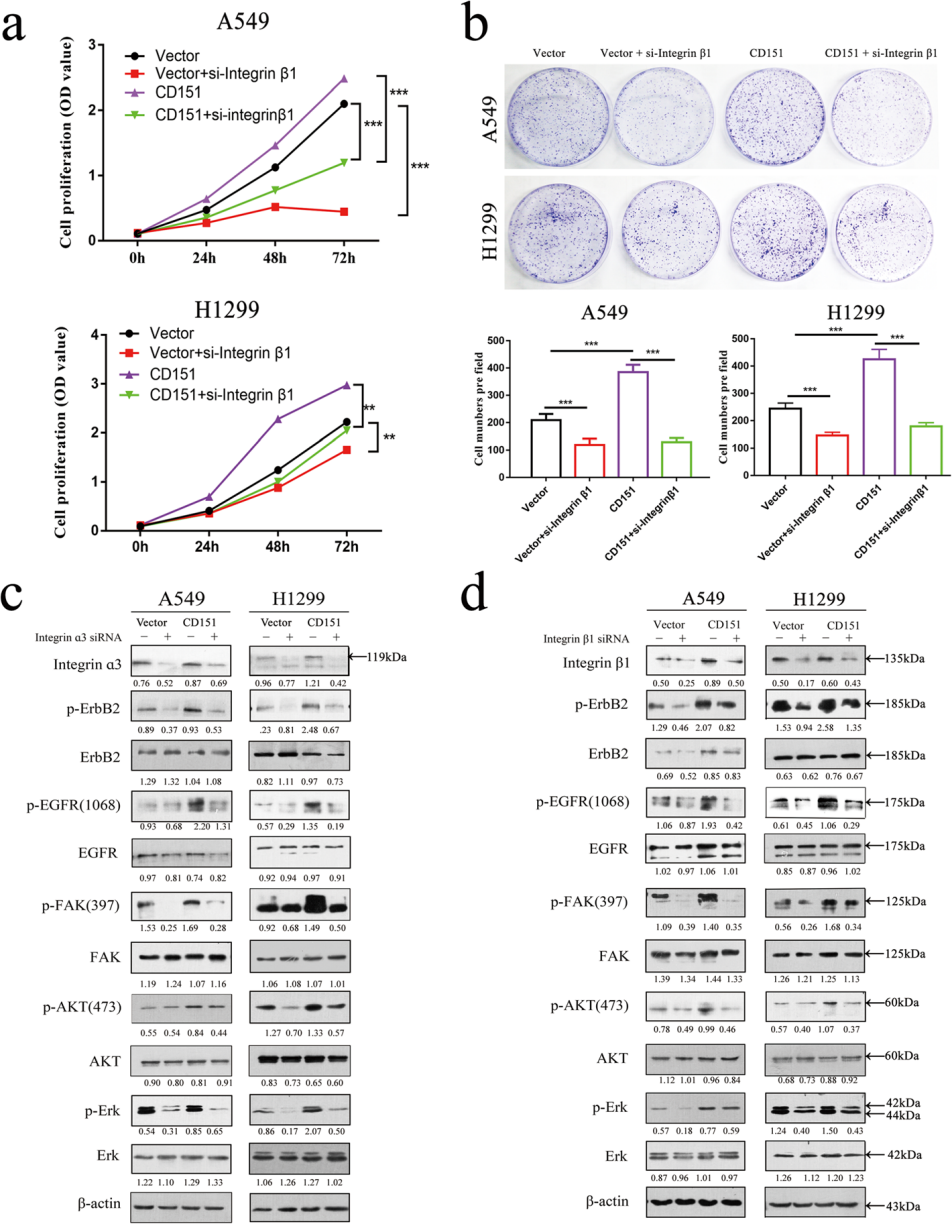
Impact of CD151 on integrin-mediated tyrosine phosphorylation cascade. **a-b** CD151-overexpressed stable A549 and H1299 cells were seeded into 6 well plates and then treated with indicated siRNA followed by CCK-8 and clone formation assays. **c-d** Effect of CD151 ablation on EGFR/ErbB2 and the downstream signaling pathways in A549 and H1299 stable cells. *P < 0.05; **P < 0.01

Based on the above, we first detected EGFR/ErbB2 and integrins expression levels in various NSCLC cell lines, which were showed in Additional file 7: Fig. S4g. Since it has been reported that integrin β1 was expressed in all histological types of lung cancer cells and increased α3β1 integrin expression in tumor cells mediates tumor proliferation and invasion [17–19]. Then we interfered integrin β1 expression using specific siRNA in CD151-overexpressed cells. As shown in Fig. 7a and b, knockdown of integrin β1 inhibited the capability of cell proliferation. However, CD151 overexpression rescued the decreased capability of cell proliferation. We next interfered integrin α3 or β1 expression in CD151 overexpressed cell lines individually, and we found that overexpression of CD151 can increase the expression of integrins, but interfering integrins rescued the increase of downstream phosphorylation protein caused by overexpression of CD151 (Fig. 7c, d). Indeed, integrins have been reported to control the EGFR signaling pathway and induce EGFR clustering, so we believe that CD151 regulates EGFR signaling pathway by controlling integrins in lung cancer [20, 21].

### CD151-EGFR signaling induced tumor growth in murine xenograft model

**Fig. 8:**
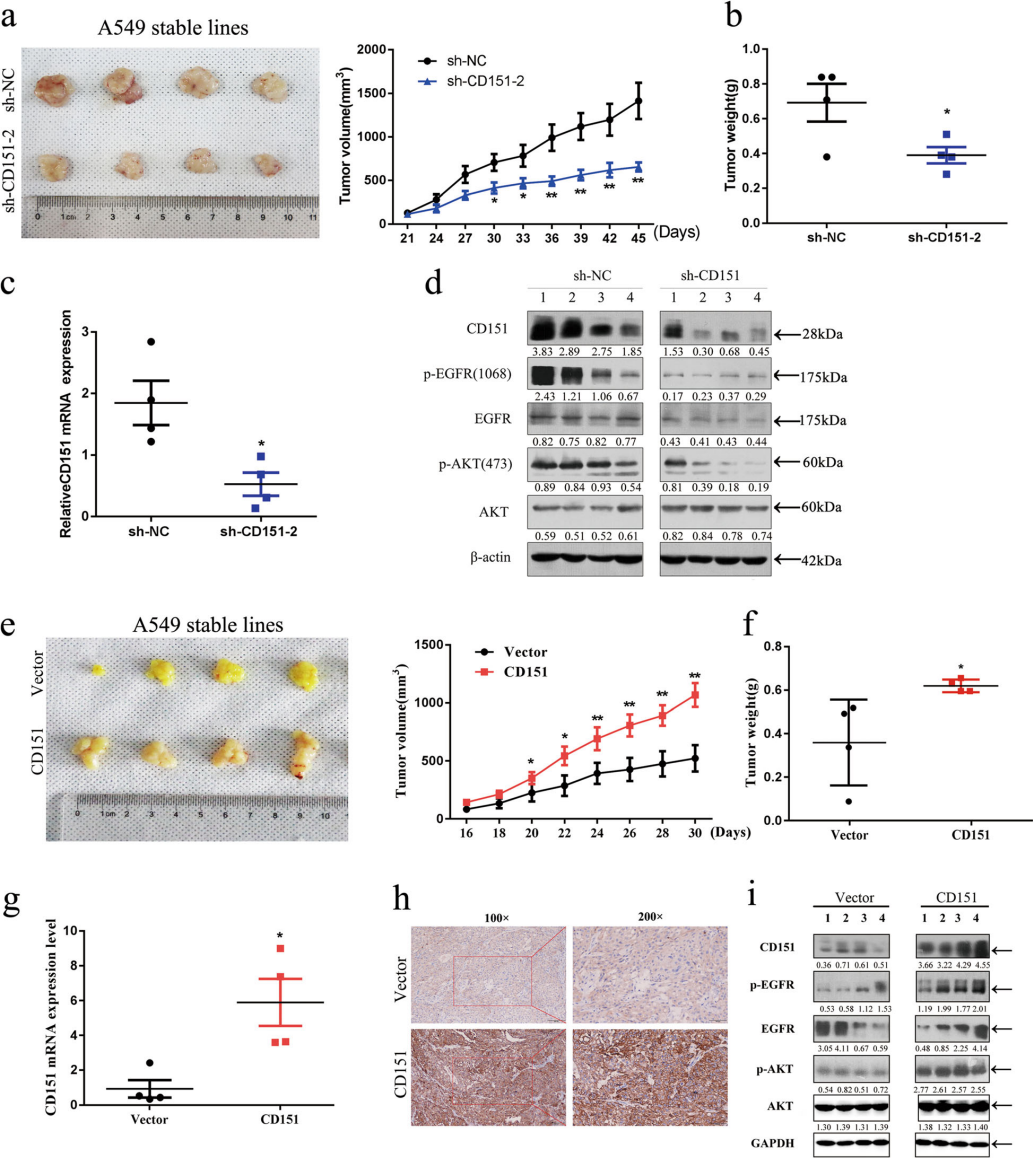
Effects of CD151 on NSCLC cell growth *in vivo*. **a** Slower growth and smaller volume of tumors were observed in nude mice subcutaneously injected with CD151-silenced A549 cells when compared with that in nude mice injected with control A549 cells (*n* = 4). **b&f** Each tumor collected from indicated mice was weighed. **c&g** CD151 mRNA expression in tumors was detected by qRT-PCR analysis. **d&i** Tumor lysates were analyzed by western blot. CD151 and EGF signaling pathways was evaluated in western blot with indicated antibodies. **e**CD151 overexpression in xenograft nude mice (*n* = 4) at the experimental endpoint; tumors were dissected and photographed as shown. **h** Immunohistochemical staining for CD151 was quantified based on staining intensity. *P < 0.05; **P < 0.01, ***P < 0.001

We then extended our study to murine subcutaneous xenograft models *in vivo*. We first used the sh-CD151-2 shRNA *in vivo* which exhibited robust knockdown efficiency in CD151 expression. Lung carcinoma xenograft mice were sacrificed after 6 weeks since inoculated with cancer cell line. As shown in Fig. 8a and b, the tumor weight and tumor volume were both decreased in CD151 knockdown group. Tissues resected from the xenograft tumors were analyzed for CD151 mRNA expression measurement (Fig. 8c). Consistently, p-EGFR and p-AKT levels were both decreased in CD151 knockdown tumors (Fig. 8d). On the other hand, overexpression of CD151 promoted tumor growth as evidenced by increased tumor volume and tumor weight (Fig. 8e, f). Both the mRNA and protein level of CD151 were increased in xenograft tumors (Fig. 8 g, h). Furthermore, we found that phosphorylation levels of EGFR and AKT were increased in CD151 overexpressed group (Fig. 8i). Collectively, these results demonstrated a crucial role of EGFR/ErbB2 signaling in CD151-mediated NSCLC cell proliferation *in vivo*.

### NSCLC cell growth inhibition induced by Gefitinib, Lapatinib, and VS6063 treatment is reversed by overexpression of CD151

**Fig. 9:**
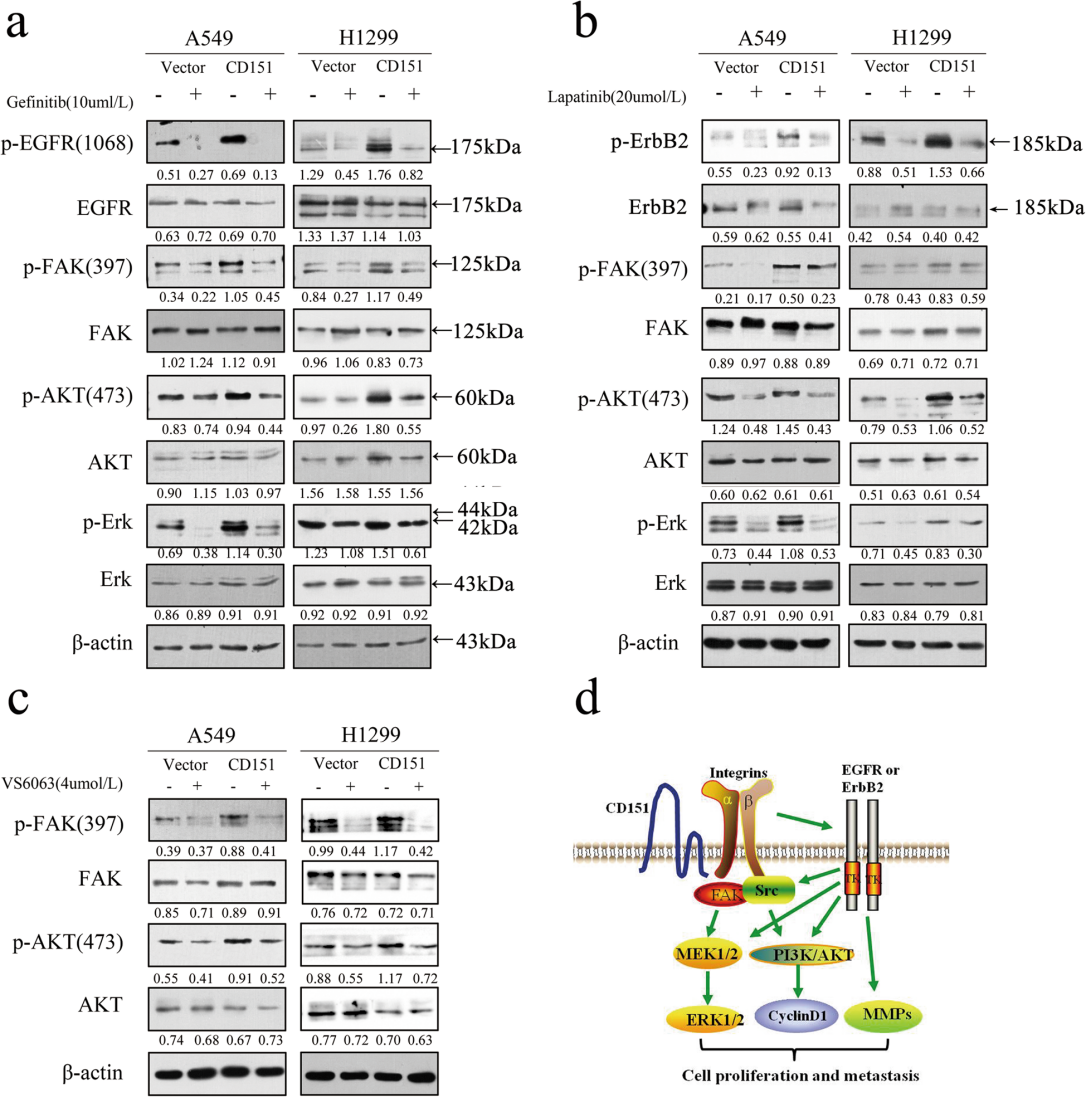
The impact of EGFR or FAK inhibition on tyrosine phosphorylation cascade. **a-c** The impact of EGFR or FAK inhibition on EGF-mediated tumor cell motility in CD151-overexpressed A549 and H1299 cells. Inhibition of EGFR or FAK was carried out by using Gefitinib or Lapatinib or TAE226 at 10 µM or 20 µM or 4 µM. **d** Schematic illustration of functional roles of CD151-α3β1 integrin complexes in NSCLC

Given the role of EGFR, ErbB2 and FAK signaling pathways in CD151-mediated NSCLC carcinogenesis, vector or CD151 transfected A549 and H1299 cells were treated with Gefitinib (EGFR-TKI inhibitor), Lapatinib (ErbB2 inhibitor) and VS6063 (FAK inhibitor) separately. These three inhibitors have been reported to inhibit proliferation of NSCLC separately [22–24]. Cell viability was assessed at 48 h after drug treatment. The results showed that overexpression of CD151 significantly reduced the sensitivity of cell lines to inhibitors (Additional file 10: Fig S6). Further, we assessed if cells are more sensitive to these inhibitors after CD151 knockdown (Additional file 11: Fig S7). Western blot assay showed that the phosphorylation levels of EGFR and downstream FAK, AKT and Erk expression level were markedly reduced compared with that in CD151 overexpressed cells in Gefitinib treatment group. In line with Gefitinib treatment, Lapatinib resulted in a large decrease of p-ErbB2, p-FAK, p-AKT and p-Erk levels in the control group over that in CD151 overexpressed cells (Fig. 9a, b). Similar phosphorylation levels of p-FAK and p-AKT were also observed when cells exposed to VS6063 (Fig. 9c). Taken together, the above findings suggested the potential of combinational CD151 knockdown therapy in improving the clinical outcome in NSCLC patients (Fig. 9d).

## Discussion

In this study, we demonstrated that CD151 plays an important role in NSCLC progression, and elevated CD151 expression indicates poor prognosis of NSCLC patients. Then we found that CD151, through binding to integrin α3β1, promotes proliferation, migration and invasion of NSCLC by activating the EGF signaling.

CD151 is involved in cell-to-cell communication, wound healing, platelet aggregation, cell trafficking and tumor progression [9]. In addition, it has been reported that CD151 is highly expressed in many solid tumors, such as breast cancer, gastric cancer and hepatocellular carcinoma [14, 25, 26]. Here, we confirmed the clinical significance of CD151 as an independent prognostic indicator of overall survival (OS) in NSCLC patients. Moreover, high CD151 protein expression retained significant prognostic prediction value both in ADC and SCC. Previous studies had demonstrated that high CD151 gene expression was significantly associated with decreased OS in Japanese and Korean cancer patients [13, 27]. However, the detailed clinical and prognostic significance of CD151 expression in Chinese NSCLC patients has not been reported yet. Here, we found that elevated CD151 level was substantially associated with increased tumor size, lymph node metastasis and high-grade tumor.

Tetraspanin CD151 plays a vital role in regulating cell adhesion through its association with laminin-binding integrins[28], the CD151-integrin association was critical for signals transduction and was the precondition for some biological effects of CD151[29–31]. Recently several findings suggested that CD151 may act as a core effector in many tumor cell types. CD151 could promotes the proliferation and migration of PC3 cells through the formation of CD151-integrin complex [32]. Moreover, CD151 has been shown to activate RhoA by facilitating integrin α3β1 and Rho controls dimerization of ErbB2, thus promoting motility and metastasis of breast cancer [16]. The involvement of CD151 due to impaired tumor-endothelial interactions was also demonstrated in CD151-knockout mice [33]. It was also reported CD151 can form complexes by interacting with integrins α3β1 and α6β1, which thus affect the biological functions of the liver cancer cells [34]. In the present study, we determined possible associations between various integrins and CD151 in the context of NSCLC, the results showed that CD151 could regulate EGFR/ErbB2 and downstream signaling proteins by interacting with integrin α3β1 complexes. However, the detailed mechanism of CD151-intergrins complex remains to be further explored.

Tetraspanin CD151 has been demonstrated to interact with EGFR/ErbB2, either directly or in conjunction with other integrin adhesion receptors [35–37]. It was reported that CD151-α3β1 integrin complex can interact with EGFR to promote tumor invasion of glioblastoma [38], and integrin-associated CD151 can promote progression and metastasis of tumor mediated by ErbB2 [36]. Focal adhesion kinase (FAK) is an important regulator of cell movement, FAK signaling can be activated via phosphorylation upon stimulation by transmembrane integrins and various growth factors, linking to the formation and turnover of focal adhesions, which then phosphorylated and induces downstream pathway signaling [39–41]. Consistently, in this report, we confirmed that CD151 could affect progression of NSCLC by regulating EGFR/ErbB2-FAK/SRC-AKT/ERK signaling pathways. Matrix metalloproteinase (MMPs) are a family of zinc endopeptidases that degrade extracellular matrix components [42], such as matrix metalloprotienases-9 (MMP9) and metalloproteinases-2 (MMP-2) can degrade various types of collagen and gelatin [43] which are closely related to the migration and invasion of tumors. In our study, we confirmed that CD151 can affect the expression of MMP2 and MMP9 by regulating EGFR/ErbB2 axis through either knockdown or overexpression of CD151, which further affect the migration and invasion of NSCLC. However, whether CD151-mediated NSCLC migration and invasion is through other independent signaling pathways rather than EGFR/ErbB2 still worth exploring.

EGFR/ErbB2 are members of the epithelial growth factor receptor family, gefitinib and lapatinib are clinically proven effective in targeting NSCLC with EGFR mutation and ErbB2 + breast cancers respectively [44–46]. In this study, A549 and H1299-CD151 overexpressed cells and control cells were treated with either gefitinib or lapatinib, results shown that there were obvious decreases in p-EGFR, p-HerB2, p-FAK, p-AKT and p-Erk levels in the control group over that in CD151 overexpressed cells, so we speculated CD151 knockdown may affect the sensitivity of NSCLC in response to anti-cancer drugs. Consistently, previous studies had showed that CD151 ablation can sensitize multiple tumor cell types to gefitinib by increasing cells apoptosis [47] and disruption of CD151 can sensitize breast cancer cells to ErbB2 inhibitors [46], this findings also confirmed our hypothesis. In addition, we used FAK inhibitor VS6063 and obtained the same results as the experiments above.

Given the role of CD151 in the development of NSCLC, CD151 may considered as a potential target for the clinical treatment of NSCLC. In view of the practical limitations of CD151 gene deletion, the most common method of targeting CD151 is through the application of CD151 monoclonal antibody (clone 9B) which can disrupt the cooperation between CD151 with integrin α6β1 [14]. Taken together, these results are worthwhile for us to further study the role of CD151 in the clinical treatment of NSCLC.

## Supplementary Information

**Additional file 1: Table S1. **Demographic and clinical characteristics and levels of CD151 protein expression in NSCLC tissue.

**Additional file 2: Table S2. **Demographic and clinical characteristics and levels of CD151 mRNA expression in NSCLC tissue.

**Additional file 3: Figure S1. **Inhibition of NSCLC cell cycle by CD151 knockout. Flow cytometry analysis of NSCLC cell lines (sh-CD151 cells vs. sh-NC cells). Cells were harvested at 72 h and stained with propidium iodide. The percentage of cells in each cell cycle phase is shown in each panel, in which the values represent the mean ± SEM of three measurements.

**Additional file 4: Figure S2. **Antibody map of the proteome profiler^TM^arrays. Adapted from the Proteome Profiler Array protocol.

**Additional file 5: Figure S3. **Human Soluble Receptor Antibody array. a-b Human Soluble Receptor Antibody array in common analytes array analysis of stable A549 cells in which CD151 either silenced or overexpressed.

**Additional file 6: Table S3. **The results of Proteome Profiler Array-Human Soluble Receptor Array Non-hematopoietic panel and Common Analytes panel. (Part N and Part C).

**Additional file 7: Figure S4. **CD151 was correlated with integrin α3/α6/β1 mRNA level in lung cancer. a-c Data obtained from TCGA database (https://portal.gdc.cancer.gov/) were analysed to explore the correlation between CD151 and integrin α3/α6/β1 mRNA levels in 103 normal tissues and 999 NSCLC tissues. d-f Data obtained from CCLE database (https://portals.broadinstitute.org/ccle) were analysed to explore the correlation between CD151 and integrin α3/α6/β1 mRNA levels in 188 lung cancer cell lines. g Total protein were extracted from several cell lines, and the expression of EGFR/ErbB2 and integrins was measured by western blotting, respectively. h Co-immunoprecipitation of CD151 and integrinβ1are shown. Protein were immunoprecipitated and detected from lysates of A549 and H1299 cells using a specific monoclonal antibody.

**Additional file 8: Figure S5. **Antibody map of the tyrosine kinase arrays. Adapted from the RayBio Human RTK Phosphorylation Antibody Array protocol.

**Additional file 9: Table S4.** List of signal densities of human RTK phosphorylation Antibody Array.

**Additional file 10: Figure S6. **Overexpression of CD151reduced the sensitivity of NSCLC cells to gefitinib, lapatinib and vs6063. The A549 and H1299stable cell lines were transfected with10 μM gefitinib (a&d), 20 μM lapatinib (b&e) or 5μM vs6063(c&f) for 48 h, respectively. After theaforementioned treatments, cell viability was assessed using CCK-8assays. The data shown represent the mean ± SD values of fourreplicate experiments. All the data were obtained from three independentexperiments and are shown as the mean ± SD values. *P<0.05; **P<0.01; ***P<0.001

**Additional file 11: Figure S7.** More sensitive to gefitinib and vs6063 after CD151 knockdown in NSCLC cells. a, b Effects of VS6063 and Gefitinib on cell cycle of vector and sh-CD151 cells. c, d The viability of vector and sh-CD151 cells after treated with VS6063 and Gefitinib was determined by CCK8 assay.

## Data Availability

Publicly data were all available in the supplementary tables. Associated analysis method was based on published articles which were cited in the references.

## Abbreviations

NSCLC: Non-small cell lung cancer
EGFR: Epidermal growth factor receptor
qRT-PCR: Quantitative reverse transcription-PCR
FAK: Focal adhesion kinase
MMPs: Matrix metalloproteinase
EMT: Epithelial-mesenchymal transition

## References

[1] Bray F, Ferlay J, Soerjomataram I, Siegel RL, Torre LA, Jemal A (2018). Global cancer statistics 2018: GLOBOCAN estimates of incidence and mortality worldwide for 36 cancers in 185 countries. Cancer J Clin.

[2] Chen W, Zheng R, Baade PD, Zhang S, Zeng H, Bray F (2016). Cancer statistics in China 2015 CA: a cancer journal for clinicians.

[3] Gould MK (2014). Clinical practice. Lung-cancer screening with low-dose computed tomography. N Engl J Med.

[4] Wu C, Li M, Meng H, Liu Y, Niu W, Zhou Y (2019). Analysis of status and countermeasures of cancer incidence and mortality in China. Science China Life sciences.

[5] Orlowski E, Chand R, Yip J, Wong C, Goschnick MW, Wright MD (2009). A platelet tetraspanin superfamily member, CD151, is required for regulation of thrombus growth and stability in vivo. Journal of thrombosis haemostasis: JTH.

[6] Bassani S, Cingolani LA, Tetraspanins (2012). Interactions and interplay with integrins. Int J Biochem Cell Biol.

[7] Robert JMH, Amoussou NG, Mai HL, et al. Tetraspanins: useful multifunction proteins for the possible design and development of small-molecule therapeutic tools. [J]. Drug Discov Today. 2021;26:56–68. 10.1016/j.drudis.2020.10.022 33137483

[8] Baleato RM, Guthrie PL, Gubler MC, Ashman LK, Roselli S (2008). Deletion of CD151 results in a strain-dependent glomerular disease due to severe alterations of the glomerular basement membrane. Am J Pathol.

[9] Sadej R, Grudowska A, Turczyk L, Kordek R, Romanska HM (2014). CD151 in cancer progression and metastasis: a complex scenario. Lab Invest.

[10] Ping Zeng Y-HW, Si M, Gu J-H, Li P, Lu P-H, Chen M-B (2017). Tetraspanin CD151 as an emerging potential poor prognostic factor across solid tumors: a systematic review and metaanalysis. Oncotarget.

[11] Kumari S, Devi Gt, Badana A, Dasari VR, Malla RR (2015). CD151-A Striking Marker for Cancer Therapy. Biomark Cancer.

[12] Hemler ME (2005). Tetraspanin functions and associated microdomains. Nat Rev Mol Cell Biol.

[13] Kwon M, Seo J, Kim Y, Kwon M, Choi J, Kim T (2013). Prognostic significance of CD151 overexpression in non-small cell lung cancer. Lung cancer.

[14] Ke AW, Shi GM, Zhou J, Huang XY, Shi YH, Ding ZB (2011). CD151 Amplifies Signaling by Integrin α6β1 to PI3K and Induces the Epithelial–Mesenchymal Transition in HCC Cells. Gastroenterology.

[15] Liu S, Tang H, Zhu J, Ding H, Zeng Y, Du W (2018). High expression of Copine 1 promotes cell growth and metastasis in human lung adenocarcinoma. Int J Oncol.

[16] Novitskaya V, Romanska H, Kordek R, Potemski P, Kusińska R, Parsons M (2014). Integrin α3β1-CD151 complex regulates dimerization of ErbB2 via RhoA. Oncogene.

[17] Guo L, Zhang F, Cai Y, Liu T (2009). Expression profiling of integrins in lung cancer cells. Pathol Res Pract.

[18] Zhou B, Gibson-Corley KN, Herndon ME, Sun Y, Gustafson-Wagner E, Teoh-Fitzgerald M (2014). Integrin alpha3beta1 can function to promote spontaneous metastasis and lung colonization of invasive breast carcinoma. Molecular cancer research: MCR.

[19] Hoshino A, Costa-Silva B, Shen TL, Rodrigues G, Hashimoto A, Tesic Mark M (2015). Tumour exosome integrins determine organotropic metastasis. Nature.

[20] Morello V, Cabodi S, Sigismund S, Camacho-Leal M, Repetto D, Volante M (2011). β1 integrin controls EGFR signaling and tumorigenic properties of lung cancer cells. Oncogene.

[21] Gilcrease M, Zhou X, Lu X, Woodward W, Hall B, Morrissey P (2009). Alpha6beta4 integrin crosslinking induces EGFR clustering and promotes EGF-mediated Rho activation in breast cancer. Journal of experimental clinical cancer research: CR.

[22] Gong H, Li Y, Yuan Y, Li W, Zhang H, Zhang Z (2020). EZH2 inhibitors reverse resistance to gefitinib in primary EGFR wild-type lung cancer cells. BMC Cancer.

[23] Zhang Y, Wang L, Sun B, Li X, Hou Q, Wang W (2020). Synthesis and Antiproliferative Activities of Novel Substituted 5-Anilino-α-Glucofuranose Derivatives. Chem Biodivers.

[24] Zhang Y, Cheng K, Xu B, Shi J, Qiang J, Shi S (2020). Epigenetic Input Dictates the Threshold of Targeting of the Integrin-Dependent Pathway in Non-small Cell Lung Cancer. Frontiers in cell developmental biology.

[25] Kwon MJ, Park S, Choi JY, Oh E, Kim YJ, Park YH (2012). Clinical significance of CD151 overexpression in subtypes of invasive breast cancer. British journal of cancer.

[26] Le Naour FAM, Greco C,Franc¸ Billard M, Sordat B. Jean-Franc¸ ois Emile, Franc¸ ois Lanza, Claude Boucheix, Eric Rubinstein. Profiling of the tetraspanin web of human colon cancer cells. Mol Cell Proteomics. 2006;5:845–57. 10.1074/mcp.M500330-MCP200 16467180

[27] Tokuhara T, Hasegawa H, Hattori N, et al. Clinical significance of CD151 gene expression in non-small cell lung cancer[J]. Clin Cancer Res. 2001;7(12):4109–14. 11751509

[28] Te ML, Juksar J, Harkes R, Wang W, Kreft M, Sonnenberg A. Tetraspanin CD151 and integrin α3β1 contribute to the stabilization of integrin α6β4-containing cell-matrix adhesions. J Cell Sci. 2019;132(19). undefined. 10.1242/jcs.235366. 10.1242/jcs.235366 31488507

[29] Zhang XA, Bontrager AL, Hemler ME (2001). Transmembrane-4 superfamily proteins associate with activated protein kinase C (PKC) and link PKC to specific beta(1) integrins. J Biol Chem.

[30] Zhang XA, Kazarov AR, Yang X, Bontrager AL, Stipp CS, Hemler ME (2002). Function of the Tetraspanin CD151–α6β1 Integrin Complex during Cellular Morphogenesis. Mol Biol Cell.

[31] Zuo H, Liu Z, Liu X, Yang J, Liu T, Wen S (2009). CD151 Gene Delivery after Myocardial Infarction Promotes Functional Neovascularization and Activates FAK Signaling. Mol Med.

[32] Wuxiao YANGPL, Jingyang LIN, Houjuan ZUO, Ping ZUO, Yuanlin ZOU, Zhengxiang LIU (2012). CD151 promotes proliferation and migration of PC3 cells via the formation of CD151-integrin α3/α6 complex. J Huazhong Univ Sci Technolog Med Sci.

[33] Takeda Y, Li Q, Kazarov AR, Epardaud M, Elpek K, Turley SJ (2011). Diminished metastasis in tetraspanin CD151-knockout mice. Blood.

[34] Fei Y, Wang J, Liu W, Zuo H, Qin J, Wang D (2012). CD151 promotes cancer cell metastasis via integrins alpha3beta1 and alpha6beta1 in vitro. Mol Med Rep.

[35] Park S-Y, Yoon S-J, Freire-de-Lima L, Kim J-H, Hakomori S-i (2009). Control of cell motility by interaction of gangliosides, tetraspanins, and epidermal growth factor receptor in A431 versus KB epidermoid tumor cells. Carbohyd Res.

[36] Deng X, Li Q, Hoff J, Novak M, Yang H, Jin H (2012). Integrin-associated CD151 drives ErbB2-evoked mammary tumor onset and metastasis. Neoplasia.

[37] Romanska HM, Potemski P, Collins SI, Williams H, Parmar S, Berditchevski F (2013). Loss of CD151/Tspan24 from the complex with integrin alpha3beta1 in invasive front of the tumour is a negative predictor of disease-free survival in oral squamous cell carcinoma. Oral Oncol.

[38] Pengcheng Z, Liu Z, Jia C, Chen Y, Xu B, Deng X (2015). CD151-α3β1 integrin complexes are prognostic markers of glioblastoma and cooperate with EGFR to drive tumor cell motility and invasion. Oncotarget.

[39] Sulzmaier FJ, Jean C, Schlaepfer DD (2014). FAK in cancer: mechanistic findings and clinical applications. Nat Rev Cancer.

[40] Kleinschmidt EG, Schlaepfer DD (2017). Focal adhesion kinase signaling in unexpected places. Curr Opin Cell Biol.

[41] Ferrer VP, Moura Neto V, Mentlein R (2018). Glioma infiltration and extracellular matrix: key players and modulators. Glia.

[42] Deryugina EI, Quigley JP (2006). Matrix metalloproteinases and tumor metastasis. Cancer Metastasis Rev.

[43] Li HC, Cao DC, Liu Y, et al. Prognostic value of matrix metalloproteinases (MMP-2 and MMP-9) in patients with lymph node-negative breast carcinoma[J]. Breast Cancer Res Treat. 2004;88(1):75–85. 10.1007/s10549-004-1200-8 15538048

[44] Fukuoka M, Wu Y-L, Thongprasert S, Sunpaweravong P, Leong S-S, Sriuranpong V (2011). Biomarker Analyses and Final Overall Survival Results From a Phase III, Randomized, Open-Label, First-Line Study of Gefitinib Versus Carboplatin/Paclitaxel in Clinically Selected Patients With Advanced Non–Small-Cell Lung Cancer in Asia (IPASS). J Clin Oncol.

[45] Inoue A, Kobayashi K, Maemondo M, Sugawara S, Oizumi S, Isobe H (2013). Updated overall survival results from a randomized phase III trial comparing gefitinib with carboplatin-paclitaxel for chemo-naive non-small cell lung cancer with sensitive EGFR gene mutations (NEJ002). Ann Oncol.

[46] Xiuwei H, Yang LMF, Li Q, Zhou P, Xu F, Krop IE. Martin E. Hemler. Disruption of Laminin-Integrin-CD151-Focal Adhesion Kinase Axis Sensitizes Breast Cancer Cells to ErbB2 Antagonists. Can Res. 2010;70:2256–63. 10.1158/0008-5472.CAN-09-4032 PMC3310185 20197472

[47] Hwang S, Takimoto T, Hemler ME (2019). Integrin-independent support of cancer drug resistance by tetraspanin CD151. Cellular and molecular life sciences. CMLS.

