# Supplementary material for: CD151 drives cancer progression depending on integrin α3β1 through EGFR signaling in non-small cell lung cancer
Source: J Exp Clin Cancer Res. 2021 Jun 9;40:192. doi: 10.1186/s13046-021-01998-4 (PMC8191020; doi:10.1186/s13046-021-01998-4)
Supplement: Supplementary file 1 — Additional file 1: Table S1. Demographic and clinical characteristics and levels of CD151 protein expression in NSCLC tissue. [file 13046_2021_1998_MOESM1_ESM.docx]

**Supplementary: ­­Table 1. Demographic and clinical characteristics of NSCLC patients and the level of CD151 protein expression in tumor tissue specimens**

| **Variables** | **Number**  **(%)** | **CD151 protein expression** | | | |
| --- | --- | --- | --- | --- | --- |
|  |  | **Low** | **High** | **χ2** | ***P*-value** |
| Gender |  |  |  |  |  |
| Male | 109(72.67) | 64 | 45 | 0.636 | 0.425 |
| Femal | 41(27.33) | 27 | 14 |  |  |
| Age |  |  |  |  |  |
| ≤ 60 year | 72(48.00) | 45 | 27 | 0.195 | 0.659 |
| >60 years | 78(52.00) | 46 | 32 |  |  |
| Histology |  |  |  |  |  |
| Squamous carcinoma | 75(50.00) | 48 | 27 | 2.009 | 0.366 |
| Adenocarinoma | 56(37.33) | 30 | 26 |  |  |
| Others | 19(12.67) | 13 | 6 |  |  |
| T status |  |  |  |  |  |
| T1 | 28(18.67) | 23 | 5 | 8.602 | **0.014** |
| T2 | 91(60.67) | 54 | 37 |  |  |
| T3-4 | 31(20.66) | 14 | 17 |  |  |
| N status |  |  |  |  |  |
| N0 | 94(62.67) | 77 | 17 | 47.639 | **<0.001** |
| N1-3 | 56(37.33) | 14 | 42 |  |  |
| Clinical stage |  |  |  |  |  |
| Ⅰ | 65(43.33) | 57 | 8 | 61.353 | **<0.001** |
| Ⅱ | 49(32.67) | 31 | 18 |  |  |
| Ⅲ | 36(24.00) | 3 | 33 |  |  |
| Pathological grade |  |  |  |  |  |
| G1 | 19(12.67) | 17 | 2 | 11.984 | **0.002** |
| G2 | 105(70.00) | 64 | 41 |  |  |
| G3 | 26(17.33) | 10 | 16 |  |  |
| P: Chi-Square Test | | | | | |
