# Supplementary material for: CD151 drives cancer progression depending on integrin α3β1 through EGFR signaling in non-small cell lung cancer
Source: J Exp Clin Cancer Res. 2021 Jun 9;40:192. doi: 10.1186/s13046-021-01998-4 (PMC8191020; doi:10.1186/s13046-021-01998-4)
Supplement: Supplementary file 2 — Additional file 2: Table S2. Demographic and clinical characteristics and levels of CD151 mRNA expression in NSCLC tissue. [file 13046_2021_1998_MOESM2_ESM.docx]

**Supplementary: Table 2. Demographic and clinical characteristics of NSCLC patients and the level of CD151 mRNA expression in tumor tissue specimens**

| **Characteristics** | **Number**(%) | **CD151 mRNA expression** | | | |
| --- | --- | --- | --- | --- | --- |
|  |  | **high** | **low** | **χ2** | ***p* value** |
| Age |  |  |  |  |  |
| ≤65 | 65(60.74) | 29 | 36 | 0.508 | 0.480 |
| >65 | 42(39.26) | 15 | 27 |  |  |
| Gender |  |  |  |  |  |
| Male | 70(65.42) | 26 | 44 | 0.891 | 0.345 |
| Female | 37(34.58) | 18 | 19 |  |  |
| Histology |  |  |  |  |  |
| Adenocarcinoma | 70(65.42) | 38 | 32 | 14.945 | **0.001** |
| Squamous cell carcinoma | 25(23.36) | 5 | 20 |  |  |
| Others | 12(11.21) | 1 | 11 |  |  |
| Degree of differentiation |  |  |  |  |  |
| Low | 38(35.31) | 14 | 24 | 0.214 | 0.644 |
| Middle | 69(64.49) | 30 | 39 |  |  |
| Smoking status |  |  |  |  |  |
| Yes | 48(44.85) | 17 | 31 | 0.782 | 0.377 |
| No | 59(55.15) | 27 | 32 |  |  |
| Clinical stage |  |  |  |  |  |
| I/II | 58(54.20) | 28 | 30 | 2.071 | 0.150 |
| III/IV | 49(45.80) | 16 | 33 |  |  |
| Distant metastasis |  |  |  |  |  |
| No | 96(89.71) | 41 | 55 | 4.783 | **0.029** |
| Yes | 11(10.29) | 3 | 8 |  |  |
| Lymph node metastasis |  |  |  |  |  |
| No | 58(54.20) | 28 | 30 | 2.071 | 0.150 |
| Yes | 49(45.80) | 16 | 33 |  |  |

P: Chi-Square Test
