# Supplementary figures and images for: CD151 drives cancer progression depending on integrin α3β1 through EGFR signaling in non-small cell lung cancer
Source: J Exp Clin Cancer Res. 2021 Jun 9;40:192. doi: 10.1186/s13046-021-01998-4 (PMC8191020; doi:10.1186/s13046-021-01998-4)

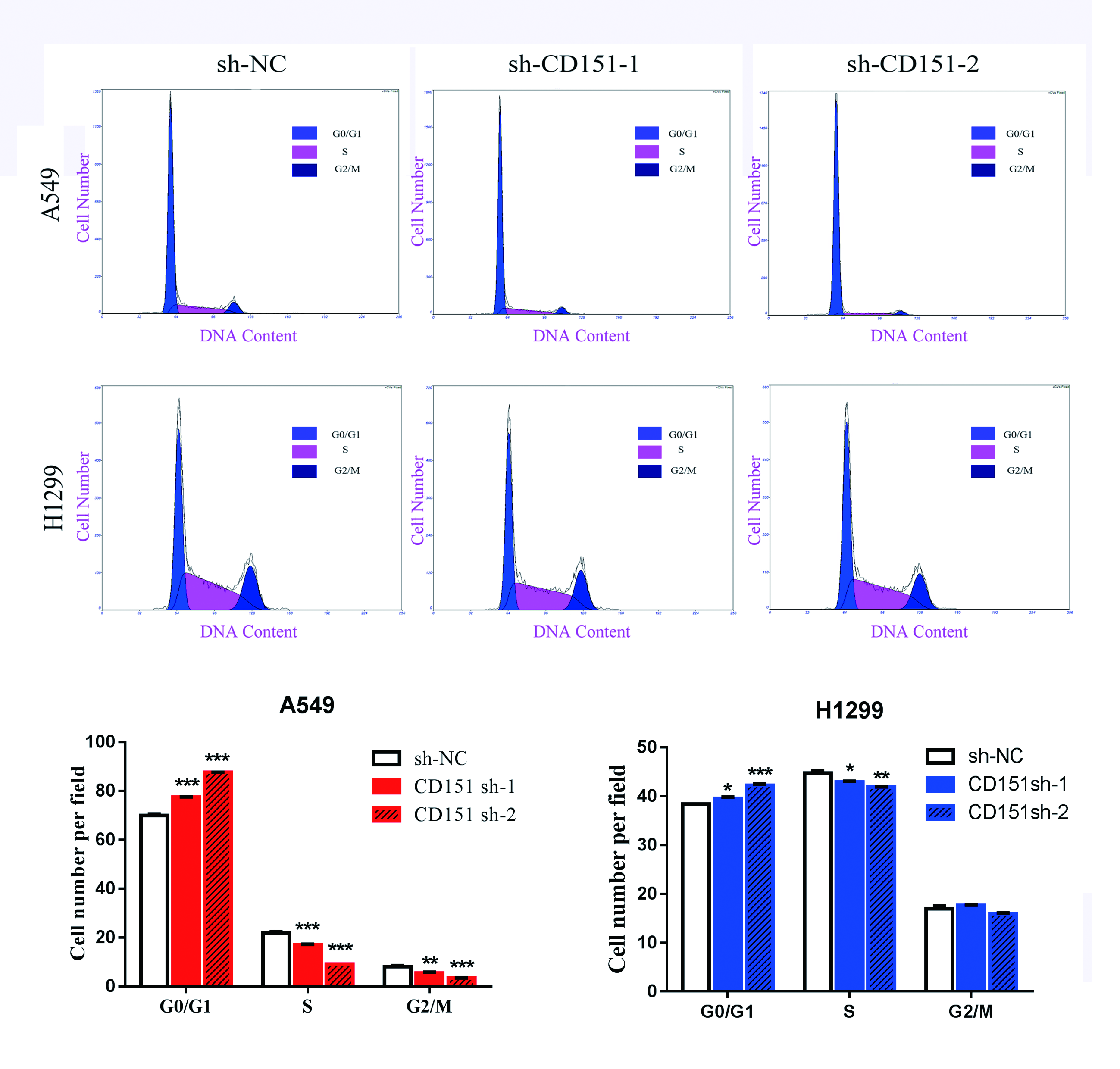

Supplement: Supplementary file 3 — Additional file 3: Figure S1. Inhibition of NSCLC cell cycle by CD151 knockout. Flow cytometry analysis of NSCLC cell lines (sh-CD151 cells vs. sh-NC cells). Cells were harvested at 72 h and stained with propidium iodide. The percentage of cells in each cell cycle phase is shown in each panel, in which the values represent the mean ± SEM of three measurements. [file 13046_2021_1998_MOESM3_ESM.tif]

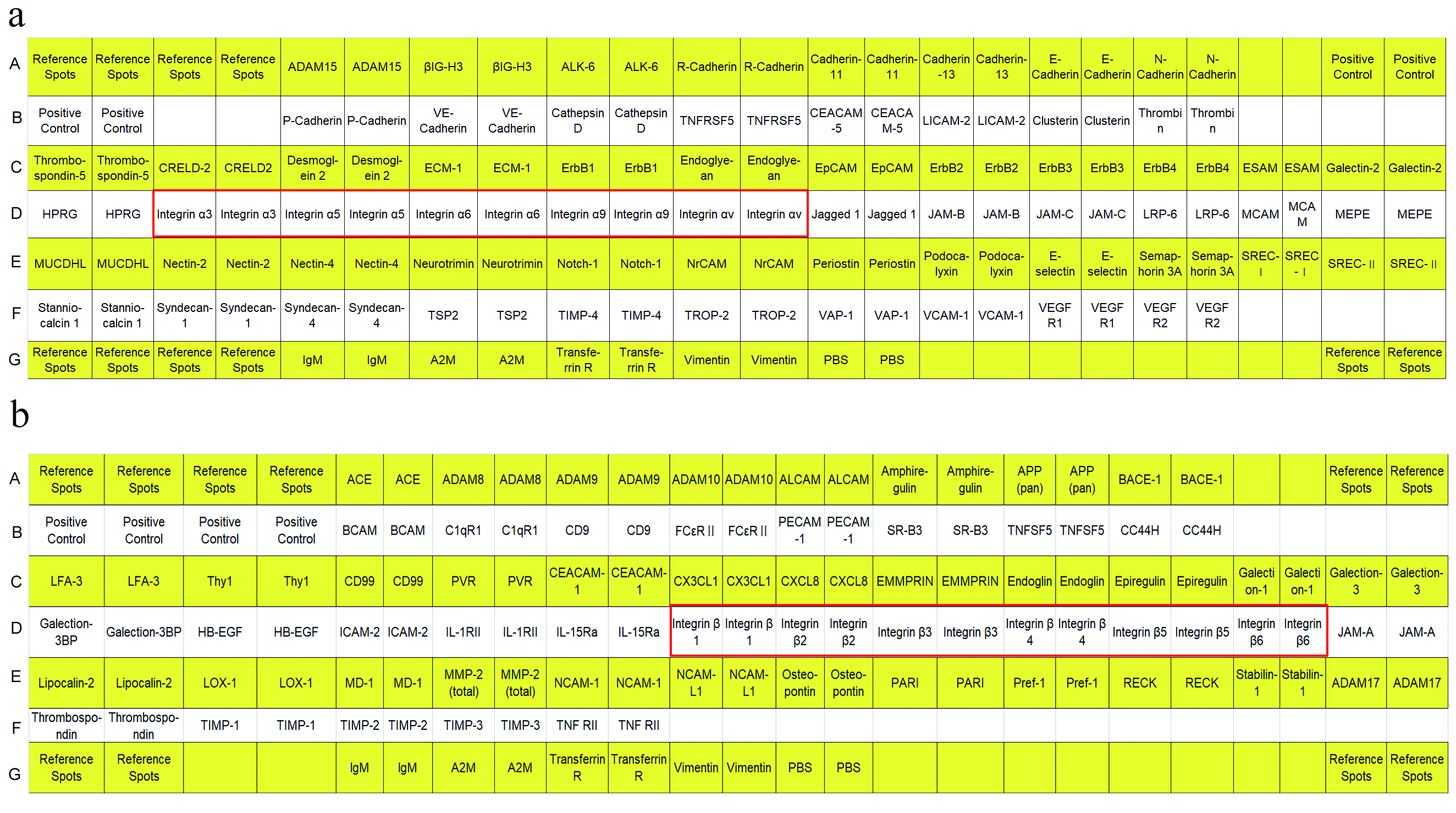

Supplement: Supplementary file 4 — Additional file 4: Figure S2. Antibody map of the proteome profilerTMarrays. Adapted from the Proteome Profiler Array protocol. [file 13046_2021_1998_MOESM4_ESM.tif]

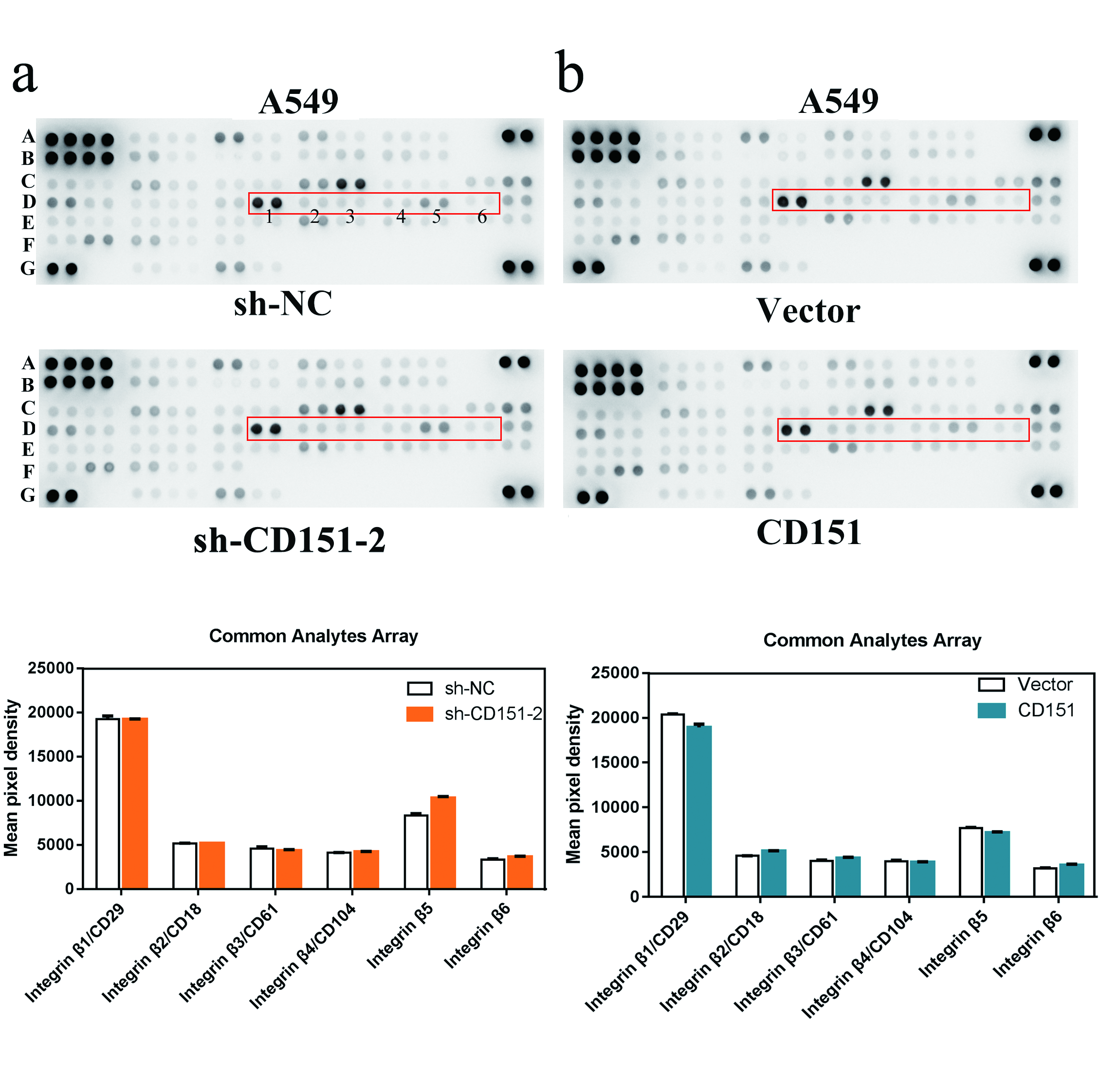

Supplement: Supplementary file 5 — Additional file 5: Figure S3. Human Soluble Receptor Antibody array. a-b Human Soluble Receptor Antibody array in common analytes array analysis of stable A549 cells in which CD151 either silenced or overexpressed. [file 13046_2021_1998_MOESM5_ESM.tif]

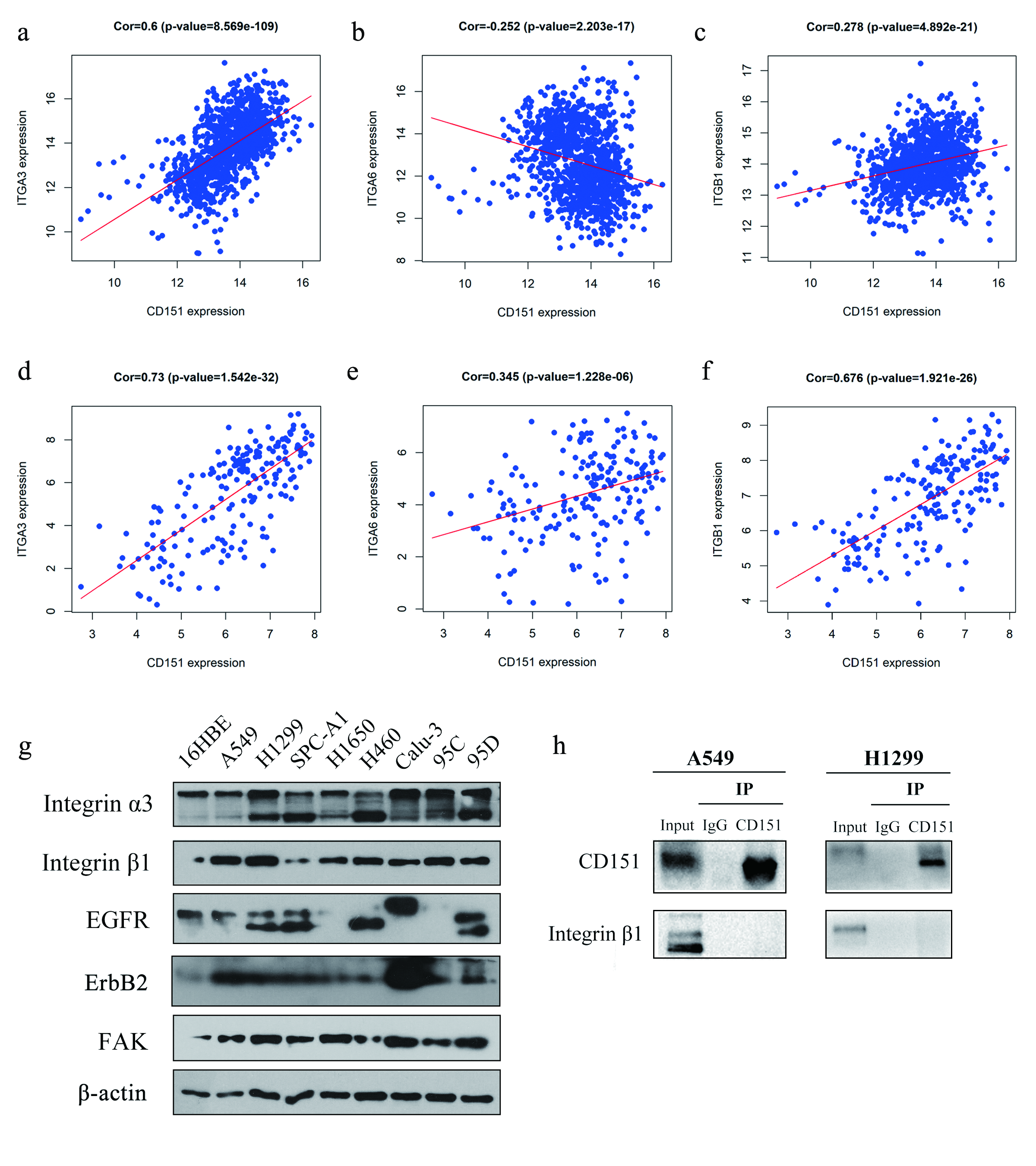

Supplement: Supplementary file 7 — Additional file 7: Figure S4. CD151 was correlated with integrin α3/α6/β1 mRNA level in lung cancer. a-c Data obtained from TCGA database (https://portal.gdc.cancer.gov/) were analysed to explore the correlation between CD151 and integrin α3/α6/β1 mRNA levels in 103 normal tissues and 999 NSCLC tissues. d-f Data obtained from CCLE database (https://portals.broadinstitute.org/ccle) were analysed to explore the correlation between CD151 and integrin α3/α6/β1 mRNA levels in 188 lung cancer cell lines. g Total protein were extracted from several cell lines, and the expression of EGFR/ErbB2 and integrins was measured by western blotting, respectively. h Co-immunoprecipitation of CD151 and integrinβ1are shown. Protein were immunoprecipitated and detected from lysates of A549 and H1299 cells using a specific monoclonal antibody. [file 13046_2021_1998_MOESM7_ESM.tif]

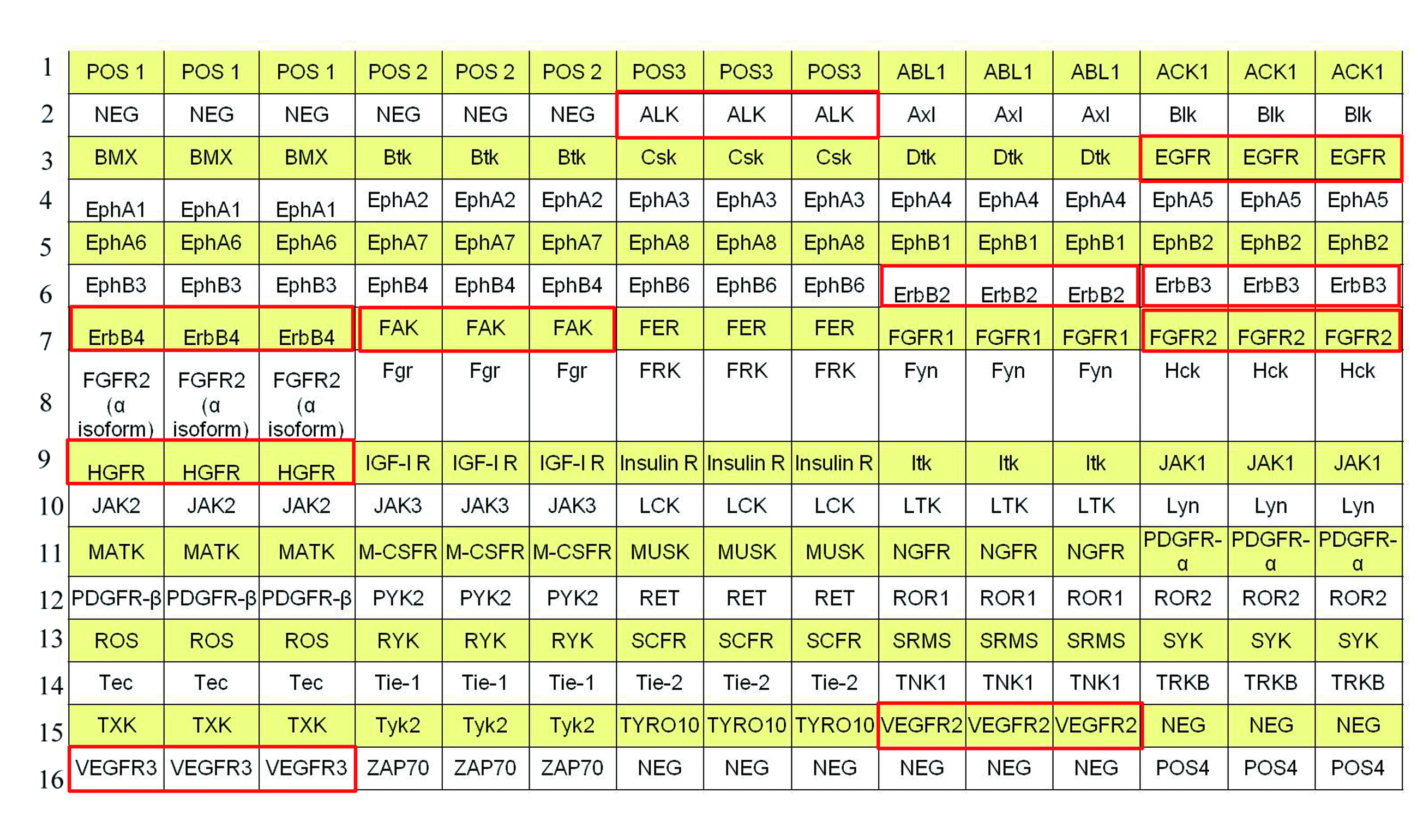

Supplement: Supplementary file 8 — Additional file 8: Figure S5. Antibody map of the tyrosine kinase arrays. Adapted from the RayBio Human RTK Phosphorylation Antibody Array protocol. [file 13046_2021_1998_MOESM8_ESM.tif]

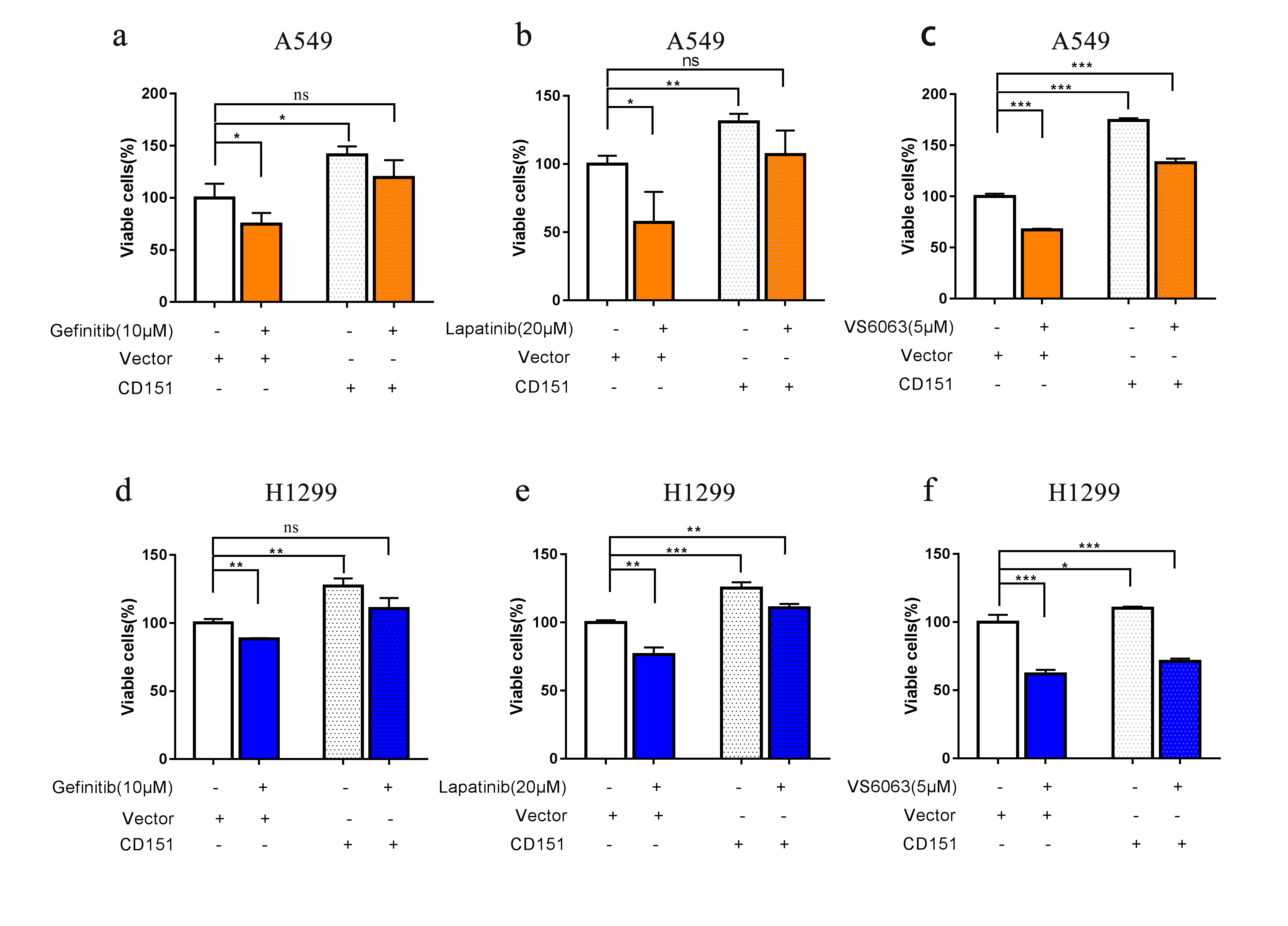

Supplement: Supplementary file 10 — Additional file 10: Figure S6. Overexpression of CD151reduced the sensitivity of NSCLC cells to gefitinib, lapatinib and vs6063. The A549 and H1299stable cell lines were transfected with10 μM gefitinib (a&d), 20 μM lapatinib (b&e) or 5μM vs6063(c&f) for 48 h, respectively. After theaforementioned treatments, cell viability was assessed using CCK-8assays. The data shown represent the mean ± SD values of fourreplicate experiments. All the data were obtained from three independentexperiments and are shown as the mean ± SD values. *P<0.05; **P<0.01; ***P<0.001 [file 13046_2021_1998_MOESM10_ESM.tif]

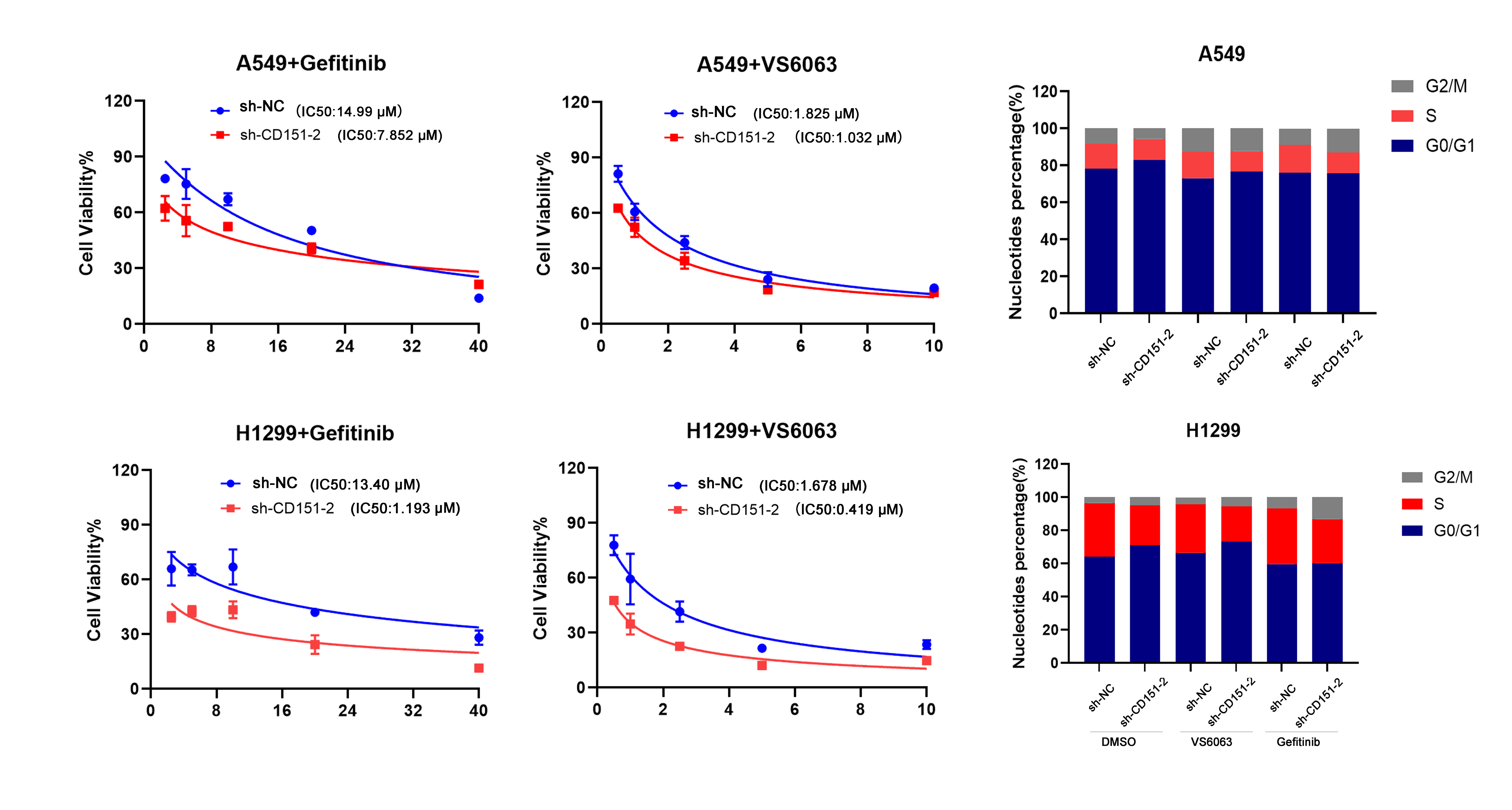

Supplement: Supplementary file 11 — Additional file 11: Figure S7. More sensitive to gefitinib and vs6063 after CD151 knockdown in NSCLC cells. a, b Effects of VS6063 and Gefitinib on cell cycle of vector and sh-CD151 cells. c, d The viability of vector and sh-CD151 cells after treated with VS6063 and Gefitinib was determined by CCK8 assay. [file 13046_2021_1998_MOESM11_ESM.tif]
