# Supplementary material for: CD151 drives cancer progression depending on integrin α3β1 through EGFR signaling in non-small cell lung cancer
Source: J Exp Clin Cancer Res. 2021 Jun 9;40:192. doi: 10.1186/s13046-021-01998-4 (PMC8191020; doi:10.1186/s13046-021-01998-4)
Supplement: Supplementary file 6 — Additional file 6: Table S3. The results of Proteome Profiler Array-Human Soluble Receptor Array Non-hematopoietic panel and Common Analytes panel. (Part N and Part C). [file 13046_2021_1998_MOESM6_ESM.docx]

**Supplementary: Table 3.The results of Proteome Profiler Array-Human Soluble Receptor Array Non-hematopoietic panel (Part N)**

| Protein | A549 | | Fold Change | A549 | | Fold Change |
| --- | --- | --- | --- | --- | --- | --- |
|  | sh-NC | sh-CD151 | sh-CD151 / sh-NC | Vector | CD151 | CD151/Vector |
| ADAM15 | 13741.32 | 9786.59 | 0.712 | 12779.93 | 16939.75 | 1.325 |
| βIG-H3 | 28322.82 | 24258.48 | 0.856 | 29516.24 | 31819.20 | 1.078 |
| BMPR-IB/ALK-6 | 8813.15 | 7217.44 | 0.818 | 8742.41 | 12028.55 | 1.375 |
| Cadherin-4/R-Cadherin | 7013.77 | 6382.52 | 0.909 | 7316.53 | 9986.75 | 1.364 |
| Cadherin-11 | 6658.12 | 5974.20 | 0.897 | 7498.85 | 9517.33 | 1.269 |
| Cadherin-13 | 7847.60 | 6881.72 | 0.876 | 7994.98 | 9619.27 | 1.203 |
| E-Cadherin | 7080.78 | 5837.00 | 0.824 | 6838.25 | 8647.15 | 1.264 |
| N-Cadherin | 6411.20 | 5272.75 | 0.822 | 6586.95 | 7740.29 | 1.175 |
| P-Cadherin | 9345.42 | 6919.93 | 0.740 | 9117.88 | 10847.40 | 1.189 |
| VE-Cadherin | 7950.96 | 7848.03 | 0.987 | 7993.67 | 12838.18 | 1.606 |
| Cathepsin D | 28375.38 | 26321.09 | 0.927 | 28899.66 | 29394.04 | 1.017 |
| CD40/TNFRSF5 | 7137.91 | 7197.58 | 1.008 | 7314.64 | 11412.96 | 1.560 |
| CEACAM-5/CD66e | 6664.14 | 6525.08 | 0.979 | 7246.13 | 7667.14 | 1.058 |
| CHL-1/L1CAM-2 | 6601.40 | 6142.06 | 0.930 | 7025.38 | 8124.52 | 1.156 |
| Clusterin | 7267.70 | 5818.93 | 0.800 | 7083.96 | 8405.27 | 1.186 |
| Coagulation Factor II/Thrombin | 4892.80 | 5020.58 | 1.026 | 5086.39 | 7209.55 | 1.417 |
| COMP/Thrombospondin-5 | 7032.76 | 6771.42 | 0.962 | 7634.26 | 11422.26 | 1.496 |
| CRELD2 | 30165.78 | 26298.53 | 0.871 | 30018.40 | 31355.80 | 1.044 |
| Desmoglein 2 | 15323.78 | 10124.82 | 0.660 | 16996.25 | 19928.28 | 1.172 |
| ECM-1 | 7436.98 | 5852.55 | 0.786 | 7704.15 | 8802.75 | 1.142 |
| EGFR/ErbB1 | 8862.42 | 7193.89 | 0.811 | 9534.30 | 11673.01 | 1.224 |
| Endoglycan | 7924.03 | 7117.36 | 0.898 | 8588.46 | 10606.72 | 1.234 |
| EpCAM/TROP-1 | 12033.04 | 11553.99 | 0.960 | 10242.75 | 11231.30 | 1.096 |
| ErbB2/HER2 | 5970.15 | 6672.47 | 1.117 | 6168.56 | 9149.96 | 1.483 |
| ErbB3/HER3 | 5547.92 | 5783.12 | 1.042 | 5701.44 | 8318.88 | 1.459 |
| ErbB4/HER4 | 5300.68 | 5951.20 | 1.122 | 5629.50 | 8437.68 | 1.498 |
| ESAM | 8244.20 | 6985.85 | 0.847 | 8764.42 | 11714.51 | 1.336 |
| Galectin-2 | 6069.02 | 5206.62 | 0.857 | 6029.93 | 8040.86 | 1.333 |
| HPRG | 6785.43 | 6184.11 | 0.911 | 7534.76 | 8917.69 | 1.183 |
| **Integrin α3/CD49c** | **10528.32** | **8215.51** | **0.780** | **10804.92** | **13678.58** | **1.265** |
| **Integrin α5/CD49e** | **11942.82** | **8575.87** | **0.718** | **11391.06** | **13598.86** | **1.193** |
| **Integrin α6/CD49f** | **7461.73** | **6113.04** | **0.819** | **7501.04** | **9284.21** | **1.237** |
| **Integrin α9** | **7690.91** | **6089.28** | **0.791** | **7655.21** | **8808.77** | **1.150** |
| **Integrin αV/CD51** | **13524.41** | **10193.81** | **0.753** | **12178.13** | **14998.68** | **1.231** |
| Jagged 1 | 6101.65 | 6433.32 | 1.054 | 6771.12 | 9257.16 | 1.367 |
| JAM-B | 6729.19 | 6046.36 | 0.898 | 7183.71 | 8931.41 | 1.243 |
| JAM-C | 27015.09 | 21664.91 | 0.801 | 25657.42 | 25774.59 | 1.004 |
| LRP-6 | 7192.33 | 7474.10 | 1.039 | 7942.86 | 11025.23 | 1.388 |
| MCAM | 9352.21 | 7265.40 | 0.776 | 10598.29 | 14146.35 | 1.334 |
| MEPE | 6167.57 | 5389.15 | 0.873 | 6106.14 | 9362.20 | 1.533 |
| MUCDHL | 7427.53 | 6007.45 | 0.808 | 7685.95 | 9713.30 | 1.263 |
| Nectin-2 | 28884.54 | 24650.37 | 0.853 | 28100.65 | 29411.56 | 1.046 |
| Nectin-4 | 8217.67 | 6526.72 | 0.794 | 8729.60 | 10700.56 | 1.225 |
| Neurotrimin | 7107.83 | 5972.67 | 0.840 | 7330.55 | 9592.88 | 1.308 |
| Notch-1 | 8969.73 | 6692.39 | 0.746 | 9183.25 | 9986.86 | 1.087 |
| NrCAM | 7203.86 | 7425.26 | 1.030 | 6981.25 | 10223.48 | 1.464 |
| Periostin | 4800.38 | 4495.10 | 0.936 | 6508.77 | 7604.07 | 1.168 |
| Podocalyxin | 7887.45 | 8146.09 | 1.032 | 8970.28 | 13201.60 | 1.471 |
| E-Selectin | 7153.60 | 5825.72 | 0.814 | 7195.98 | 8193.28 | 1.138 |
| Semaphorin 3A | 7024.61 | 7180.97 | 1.022 | 7952.06 | 12338.65 | 1.551 |
| SREC-I | 8240.69 | 7795.699 | 0.945 | 8893.74 | 13768.91 | 1.548 |
| SREC-II | 6892.67 | 5884.41 | 0.853 | 6956.17 | 10321.04 | 1.483 |
| Stanniocalcin 1 | 8567.11 | 6597.46 | 0.770 | 7732.81 | 10973.21 | 1.419 |
| Syndecan-1 | 12066.11 | 9652.23 | 0.799 | 13307.93 | 17685.65 | 1.328 |
| Syndecan-4 | 9012.54 | 6991.32 | 0.775 | 9924.33 | 11367.95 | 1.145 |
| Thrombospondin-2 | 5119.46 | 5095.59 | 0.995 | 5323.56 | 7713.35 | 1.448 |
| TIMP-4 | 5888.35 | 5784.88 | 0.982 | 6140.09 | 8608.94 | 1.402 |
| TROP-2 | 6916.43 | 6133.74 | 0.886 | 7337.88 | 9628.69 | 1.312 |
| VAP-1 | 6692.51 | 6272.58 | 0.937 | 7972.32 | 9925.76 | 1.245 |
| VCAM-1 | 6671.15 | 5579.35 | 0.836 | 6937.78 | 8137.22 | 1.172 |
| VEGF-R1 | 5911.35 | 5898.86 | 0.997 | 6339.70 | 9082.84 | 1.432 |
| VEGF-R2 | 5651.40 | 4996.16 | 0.884 | 5853.86 | 7210.10 | 1.231 |

**The results of Proteome Profiler Array-Human Soluble Receptor Array Common Analytes panel (Part C)**

| Protein | A549 | | Fold Change | A549 | | Fold Change |
| --- | --- | --- | --- | --- | --- | --- |
|  | sh-NC | sh-CD151 | sh-CD151 / sh-NC | Vector | CD151 | CD151/  Vector |
| ACE | 4137.95 | 4123.14 | 0.996 | 4127.86 | 4075.32 | 0.987 |
| ADAM8 | 3503.67 | 3508.19 | 1.001 | 3086.52 | 3282.01 | 1.063 |
| ADAM9 | 12073.89 | 10874.48 | 0.901 | 9060.30 | 9173.15 | 1.012 |
| ADAM10 | 3315.30 | 3261.71 | 0.984 | 2765.95 | 3107.58 | 1.124 |
| ALCAM/CD166 | 6915.75 | 6765.76 | 0.978 | 5978.40 | 6535.44 | 1.093 |
| Amphiregulin | 3253.53 | 3640.38 | 1.119 | 3171.56 | 3783.05 | 1.193 |
| APP(pan) | 3530.51 | 3719.46 | 1.054 | 3263.25 | 3383.80 | 1.037 |
| BACE-1 | 2971.64 | 3310.49 | 1.114 | 2875.34 | 2919.79 | 1.015 |
| BCAM | 6847.24 | 6514.75 | 0.951 | 6078.27 | 5581.43 | 0.918 |
| C1qR1/CD93 | 3152.90 | 3457.68 | 1.097 | 2964.24 | 3310.39 | 1.117 |
| CD9 | 2674.46 | 2691.10 | 1.006 | 2287.23 | 2413.74 | 1.055 |
| CD23/FcεRII | 3293.84 | 3317.41 | 1.007 | 3124.52 | 3315.68 | 1.061 |
| CD31/PECAM-1 | 4064.65 | 4037.81 | 0.993 | 3605.07 | 3872.72 | 1.074 |
| CD36/SR-B3 | 5164.78 | 5748.57 | 1.113 | 5135.63 | 5969.65 | 1.162 |
| CD40Ligand/TNFSF5 | 5192.41 | 5660.10 | 1.090 | 4546.88 | 4919.47 | 1.082 |
| CD44H | 3999.72 | 4345.32 | 1.086 | 3697.39 | 3873.21 | 1.048 |
| CD58/LFA-3 | 6883.81 | 6366.79 | 0.925 | 6564.02 | 7751.98 | 1.181 |
| CD90/Thy1 | 4998.82 | 4916.18 | 0.983 | 4808.33 | 6027.37 | 1.254 |
| CD99 | 7475.19 | 7788.44 | 1.042 | 7194.27 | 7205.04 | 1.001 |
| CD155/PVR | 3725.23 | 4191.55 | 1.125 | 3803.35 | 4188.86 | 1.101 |
| CEACAM-1/CD66a | 3463.26 | 3757.08 | 1.085 | 3641.25 | 3714.17 | 1.020 |
| CX3CL1/Fractalkine | 3792.10 | 3993.07 | 1.053 | 4001.64 | 4091.40 | 1.022 |
| CXCL8/IL-8 | 9530.85 | 10138.40 | 1.064 | 4685.38 | 5382.21 | 1.149 |
| EMMPRIN/CD147 | 17591.85 | 17650.06 | 1.003 | 16638.53 | 17151.89 | 1.031 |
| Endoglin/CD105 | 3202.35 | 3789.31 | 1.183 | 3548.21 | 3535.03 | 0.996 |
| Epiregulin | 2831.37 | 3725.14 | 1.316 | 3198.60 | 3263.15 | 1.020 |
| Galectin-1 | 4891.17 | 5316.98 | 1.087 | 5501.89 | 5027.69 | 0.914 |
| Galectin-3 | 10798.76 | 11998.56 | 1.111 | 11639.04 | 11638.65 | 1.000 |
| Galectin-3BP/MAC-2BP | 12163.17 | 9949.06 | 0.818 | 9797.25 | 11258.25 | 1.149 |
| HB-EGF | 5538.16 | 5337.47 | 0.964 | 5049.43 | 5612.53 | 1.112 |
| ICAM-2/CD102 | 3664.53 | 3901.77 | 1.065 | 3828.56 | 3991.73 | 1.043 |
| IL-1RII | 3094.21 | 3594.49 | 1.162 | 3262.48 | 3637.69 | 1.115 |
| IL-15Rα | 5070.98 | 5327.08 | 1.051 | 5393.56 | 5307.07 | 0.984 |
| Integrinβ1/CD29 | 19262.30 | 19229.87 | 0.998 | 20370.70 | 18954.14 | 0.930 |
| Integrinβ2/CD18 | 5177.48 | 5174.69 | 0.999 | 4585.04 | 5131.01 | 1.119 |
| Integrinβ3/CD61 | 4598.99 | 4361.84 | 0.948 | 4026.56 | 4313.25 | 1.071 |
| Integrinβ4/CD104 | 4117.57 | 4208.48 | 1.022 | 3966.71 | 3883.11 | 0.979 |
| Integrinβ5 | 8324.90 | 10327.93 | 1.241 | 7684.44 | 7166.85 | 0.933 |
| Integrinβ6 | 3336.85 | 3644.71 | 1.092 | 3200.71 | 3522.43 | 1.101 |
| JAM-A | 8063.40 | 8297.19 | 1.029 | 7634.13 | 8755.71 | 1.147 |
| Lipocalin-2/NGAL | 5016.24 | 4419.56 | 0.881 | 4401.96 | 5038.94 | 1.145 |
| LOX-1/SR-E1 | 3702.82 | 4034.93 | 1.090 | 3934.77 | 4786.69 | 1.217 |
| MD-1 | 3467.98 | 3678.38 | 1.061 | 3765.83 | 3910.91 | 1.039 |
| MMP-2(total) | 3318.18 | 3518.87 | 1.060 | 3546.29 | 3657.41 | 1.031 |
| NCAM-1/CD56 | 3640.67 | 3830.87 | 1.052 | 3827.41 | 3853.00 | 1.007 |
| NCAM-L1 | 4242.73 | 4191.94 | 0.988 | 4153.45 | 3974.70 | 0.957 |
| Osteopontin | 8824.69 | 7911.01 | 0.896 | 8051.96 | 8743.11 | 1.086 |
| PAR1 | 3906.59 | 3877.53 | 0.993 | 3559.47 | 3748.23 | 1.053 |
| Pref-1/DLK-1/FA1 | 4112.76 | 4813.53 | 1.170 | 4530.30 | 4566.37 | 1.008 |
| RECK | 3529.16 | 4126.13 | 1.169 | 3489.53 | 3711.77 | 1.064 |
| Stabilin-1 | 3116.53 | 3420.45 | 1.098 | 3030.72 | 3326.74 | 1.098 |
| TACE/ADAM17 | 4837.87 | 5360.47 | 1.108 | 4483.15 | 5663.33 | 1.263 |
| Thrombospondin | 4892.52 | 5166.32 | 1.056 | 5430.22 | 6368.81 | 1.173 |
| TIMP-1 | 9696.71 | 9910.48 | 1.022 | 9791.38 | 12251.11 | 1.251 |
| TIMP-2 | 6473.87 | 6223.73 | 0.961 | 6517.16 | 6676.29 | 1.024 |
| TIMP-3 | 3063.71 | 3159.92 | 1.031 | 3126.73 | 3311.93 | 1.059 |
| TNFRII/TNFRSF1B | 3598.82 | 3852.32 | 1.070 | 3770.84 | 3880.13 | 1.029 |
