# Supplementary material for: CD151 drives cancer progression depending on integrin α3β1 through EGFR signaling in non-small cell lung cancer
Source: J Exp Clin Cancer Res. 2021 Jun 9;40:192. doi: 10.1186/s13046-021-01998-4 (PMC8191020; doi:10.1186/s13046-021-01998-4)
Supplement: Supplementary file 9 — Additional file 9: Table S4. List of signal densities of human RTK phosphorylation Antibody Array. [file 13046_2021_1998_MOESM9_ESM.docx]

**Supplementary: Table 4. List of signal densities of human RTK phosphorylation Antibody Array**

| RTKs List | A549 | | Fold Change | A549 | | Fold Change |
| --- | --- | --- | --- | --- | --- | --- |
|  | sh-NC | sh-CD151 | sh-CD151 / sh-NC | Vector | CD151 | CD151/Vector |
| ABL1 | 770 | 735 | 0.955 | 784 | 834 | 1.064 |
| ACK1 | 868 | 753 | 0.867 | 804 | 855 | 1.063 |
| ALK | 1,066 | 1,069 | 1.002 | 1,118 | 1,125 | 1.007 |
| Axl | 753 | 530 | 0.705 | 652 | 755 | 1.158 |
| Blk | 111 | 138 | 1.24 | 216 | 167 | 0.770 |
| BMX | 626 | 539 | 0.861 | 358 | 492 | 1.377 |
| Btk | 423 | 405 | 0.956 | 344 | 490 | 1.426 |
| Csk | 145 | 185 | 1.276 | 189 | 986 | 5.229 |
| Dtk | 2 | 41 | 17.602 | 10 | 3 | 0.335 |
| **EGFR** | **476** | **400** | **0.841** | **363** | **505** | **1.391** |
| EphA1 | 74 | 100 | 1.357 | 23 | 110 | 4.831 |
| EphA2 | 631 | 565 | 0.896 | 734 | 802 | 1.094 |
| EphA3 | 1,007 | 1,112 | 1.105 | 1,059 | 1,178 | 1.113 |
| EphA4 | 933 | 784 | 0.841 | 848 | 885 | 1.044 |
| EphA5 | 844 | 849 | 1.006 | 905 | 964 | 1.064 |
| EphA6 | 732 | 883 | 1.207 | 774 | 769 | 0.994 |
| EphA7 | 670 | 630 | 0.941 | 745 | 762 | 1.021 |
| EphA8 | 446 | 354 | 0.795 | 439 | 578 | 1.317 |
| EphB1 | 677 | 769 | 1.136 | 602 | 605 | 1.005 |
| EphB2 | 801 | 823 | 1.027 | 900 | 905 | 1.007 |
| EphB3 | 1,718 | 1,927 | 1.122 | 1,630 | 1,911 | 1.172 |
| EphB5 | 761 | 702 | 0.922 | 656 | 696 | 1.062 |
| EphB6 | 1,409 | 1,480 | 1.051 | 1,505 | 1,584 | 1.052 |
| **ErbB2** | **166** | **140** | **0.844** | **139** | **228** | **1.638** |
| ErbB3 | 470 | 466 | 0.992 | 353 | 378 | 1.071 |
| ErbB4 | 93 | 51 | 0.554 | 125 | 129 | 1.033 |
| FAK | 532 | 495 | 0.929 | 575 | 592 | 1.030 |
| FER | 481 | 741 | 1.540 | 594 | 1,735 | 2.923 |
| FGFR1 | 19,293 | 23,814 | 1.234 | 24,482 | 18,042 | 0.736 |
| FGFR2 | 167 | 165 | 0.990 | 160 | 143 | 0.890 |
| FGFR2 (α isoform) | 104 | 92 | 0.885 | 111 | 105 | 0.949 |
| Fgr | 739 | 756 | 1.022 | 734 | 716 | 0.974 |
| FRK | 788 | 665 | 0.844 | 805 | 847 | 1.052 |
| Fyn | 2,177 | 2,599 | 1.194 | 2,300 | 2,308 | 1.003 |
| Hck | 639 | 609 | 0.953 | 525 | 627 | 1.193 |
| HGFR | 1,078 | 1,057 | 0.980 | 1,236 | 1,234 | 0.998 |
| IGF-I R | 98 | 142 | 1.445 | 165 | 110 | 0.663 |
| Insulin R | 666 | 623 | 0.936 | 737 | 658 | 0.892 |
| Itk | 952 | 1,190 | 1.251 | 1,015 | 1,040 | 1.024 |
| JAK1 | 652 | 666 | 1.020 | 589 | 759 | 1.288 |
| JAK2 | 431 | 788 | 1.827 | 399 | 589 | 1.476 |
| JAK3 | 1,366 | 1,591 | 1.165 | 1,723 | 2,103 | 1.220 |
| LCK | 804 | 865 | 1.075 | 902 | 844 | 0.935 |
| LTK | 1,024 | 1,080 | 1.055 | 1,009 | 1,153 | 1.142 |
| Lyn | 1,534 | 1,644 | 1.071 | 1,608 | 1,810 | 1.125 |
| MATK | 1,135 | 1,387 | 1.221 | 1,298 | 1,550 | 1.194 |
| M-CSFR | 29 | 0 | 0 | 81 | 62 | 0.767 |
| MUSK | 781 | 872 | 1.115 | 1,037 | 953 | 0.919 |
| NGFR | 142 | 251 | 1.774 | 226 | 252 | 1.117 |
| PDGFR-α | 1,557 | 1,839 | 1.181 | 1,547 | 1,678 | 1.085 |
| PDGFR-β | 276 | 272 | 0.986 | 323 | 370 | 1.143 |
| PYK2 | 276 | 364 | 1.320 | 302 | 307 | 1.015 |
| RET | 0 | 0 | 0 | 0 | 2 | 13.609 |
| ROR1 | 151 | 72 | 0.476 | 235 | 195 | 0.831 |
| ROR2 | 448 | 1,131 | 2.523 | 499 | 397 | 0.794 |
| ROS | 499 | 503 | 1.009 | 659 | 564 | 0.856 |
| RYK | 464 | 626 | 1.349 | 782 | 573 | 0.732 |
| SCFR | 101 | 183 | 1.805 | 201 | 132 | 0.654 |
| SRMS | 500 | 547 | 1.093 | 498 | 483 | 0.970 |
| SYK | 520 | 559 | 1.075 | 536 | 502 | 0.936 |
| Tec | 1,204 | 1,299 | 1.078 | 1,158 | 1,182 | 1.020 |
| Tie-1 | 351 | 264 | 0.751 | 407 | 294 | 0.722 |
| Tie-2 | 61 | 4 | 0.060 | 96 | 98 | 1.018 |
| TNK1 | 0 | 0 | 0 | 0 | 0 | 0 |
| TRKB | 959 | 1,012 | 1.055 | 1,078 | 992 | 0.920 |
| TXK | 874 | 924 | 1.057 | 973 | 990 | 1.017 |
| Tyk2 | 254 | 316 | 1.243 | 271 | 221 | 0.814 |
| TYRO10 | 752 | 895 | 1.190 | 882 | 851 | 0.965 |
| VEGFR2 | 7 | 1 | 0.099 | 62 | 33 | 0.531 |
| VEGFR3 | 475 | 591 | 1.244 | 590 | 704 | 1.193 |
| ZAP70 | 806 | 838 | 1.040 | 880 | 847 | 0.963 |
